# Synthetic development of a broadly neutralizing antibody against snake venom long-chain α-neurotoxins

**DOI:** 10.1126/scitranslmed.adk1867

**Published:** 2024-02-21

**Authors:** Irene S. Khalek, R. R. Senji Laxme, Yen Thi Kim Nguyen, Suyog Khochare, Rohit N. Patel, Jordan Woehl, Jessica M. Smith, Karen Saye-Francisco, Yoojin Kim, Laetitia Misson Mindrebo, Quoc Tran, Mateusz Kędzior, Evy Boré, Oliver Limbo, Megan Verma, Robyn L. Stanfield, Stefanie K. Menzies, Stuart Ainsworth, Robert A. Harrison, Dennis R. Burton, Devin Sok, Ian A. Wilson, Nicholas R. Casewell, Kartik Sunagar, Joseph G. Jardine

**Affiliations:** 1 Department of Immunology and Microbiology, Scripps Research Institute, La Jolla, CA 92037, USA; 2 IAVI Neutralizing Antibody Center, Scripps Research Institute, La Jolla, CA 92037, USA; 3 IAVI, New York, NY 10004, USA; 4 Evolutionary Venomics Lab, Centre for Ecological Sciences, Indian Institute of Science, Bangalore 560012, Karnataka, India; 5 Department of Integrative Structural and computational Biology, Scripps Research Institute, La Jolla, CA 92037, USA; 6 Centre for Snakebite Research & Interventions, Department of Tropical Disease Biology, Liverpool School of Tropical Medicine, Liverpool L3 5QA, UK; 7 Consortium for HIV/AIDS Vaccine Development (CHAVD), Scripps Research Institute, La Jolla, CA 92037, USA; 8 Ragon Institute of Massachusetts General Hospital, Massachusetts Institute of Technology, and Harvard University, Cambridge, MA 02139, USA; 9 Skaggs Institute for Chemical Biology, Scripps Research Institute, La Jolla, CA 92037, USA

## Abstract

Snakebite envenoming is a major global public health concern for which improved therapies are urgently needed. The antigenic diversity present in snake venom toxins from various species presents a considerable challenge to the development of a universal antivenom. Here, we used a synthetic human antibody library to find and develop an antibody that neutralizes long-chain three-finger α-neurotoxins produced by numerous medically relevant snakes. Our antibody bound diverse toxin variants with high affinity, blocked toxin binding to the nicotinic acetyl-choline receptor in vitro, and protected mice from lethal venom challenge. Structural analysis of the antibody-toxin complex revealed a binding mode that mimics the receptor-toxin interaction. The overall workflow presented is generalizable for the development of antibodies that target conserved epitopes among antigenically diverse targets, and it offers a promising framework for the creation of a monoclonal antibody–based universal antivenom to treat snakebite envenoming.

## Introduction

Snakebite envenoming is estimated to cause 81,000 to 138,000 deaths annually, with an additional 400,000 or more people left with permanent disabilities (1). The impact is particularly severe in low- and middle-income countries in sub-Saharan Africa and Asia because of the alarmingly large number of snakebites and limited access to adequate medical resources. These substantial public health costs warranted the designation of snakebite as a neglected tropical disease by the World Health Organization (WHO) in 2017 (2). Animal-derived polyclonal antibody–based antivenom therapy is the primary medical countermeasure to treat snakebites and has been in use for over a century. The currently marketed antivenoms are produced by the hyper-immunization of large animals, such as equines and ovines, until they produce a robust antibody response. From there, polyclonal immunoglobulins (IgGs) are purified, sometimes processed into antibody fragments [F(ab′)_2_ or Fabs], and formulated for intravenous delivery to snakebite victims (3). A high-quality antivenom matched to the biting snake species is effective at reducing morbidity and mortality (4). However, in practice, antivenoms often have substantial problems with safety, efficacy, potency, cost, and distribution (5, 6). The immunogenicity of heterologous proteins and impurities present in antivenoms can induce serum sickness and severe anaphylaxis (7). Furthermore, only a fraction of the antibodies in antivenoms therapeutically target the venom toxins, thus requiring large doses of a product with high batch-to-batch variability to effect cure (8, 9).

The development of an effective antivenom is challenging, because venoms are complex mixtures with multiple classes and isoforms of toxic proteins that exhibit highly diverse structures, functions, biological targets, and compositions in different snake species (1, 10). Owing to their clinical relevance, snake venom toxins have been extensively studied, and their compositional diversity has been well characterized. The major snake venom toxin families include phos-pholipases, metalloproteases, three-finger toxins (3FTxs), and serine proteases (11). Several other ancillary toxins families, including cysteine-rich secretory proteins, hyaluronidases, nucleases, Kunitz-type serine protease inhibitors, and C-type lectins, have also been described. However, studies have highlighted that inhibiting or neutralizing the major toxin families can alleviate venom-associated mortality and morbidity in animal models (12, 13). 3FTxs are among the most abundant and lethal toxins present in the venom of elapids (14), a major medically relevant family of snakes that includes cobras, kraits, and mambas. Despite their structural similarity, 3FTxs exhibit extensive antigenic and functional diversity and are categorized into various subfamilies (15). The α-neurotoxin class of 3FTx targets the muscle-type nicotinic acetylcholine receptor (nAChR) at neuromuscular junctions, leading to paralysis and death by asphyxiation (16). The α-neurotoxins are further categorized into short-chain (3FTx-S) and long-chain (3FTx-L) variants on the basis of their sequence length (15). Because α-neurotoxins are abundantly produced by a wide range of elapids, they are considered key targets for antivenom development (16).

In practice, toxin variant diversity has resulted in antivenoms that are relatively species-specific, prompting manufacturers to immunize animals with venoms from multiple species of medically relevant snakes to expand the functional breadth of coverage (10), but, in doing so, generating a need for higher therapeutic doses due to reduced dose efficacy (4). Many of the current safety, efficacy, and reproducibility limitations with animal-derived polyclonal antivenoms could be addressed with the development of a recombinant antibody cocktail (17). Of late, several studies have demonstrated the effectiveness of monoclonal antibodies (mAbs) or single-domain antibodies (sdAbs) in neutralizing a particular toxin to protect envenomed experimental animals (18–25). However, like the animal-derived polyclonal antivenoms, nearly all mAbs and sdAbs reported to date have a limited breadth of coverage outside the initial target variant. A broad-spectrum recombinant antivenom would require an unfeasible number of these antibodies to cover the multiple toxin variants across diverse snake species.

Although toxins within a class exhibit considerable antigenic diversity, the active region of the protein is often highly conserved to preserve functionality (26). We hypothesized that the development of antibodies targeting these functionally conserved regions could achieve two objectives: (i) broad recognition across a toxin class and (ii) disruption of toxin function. Broadly neutralizing antibodies (bnAbs) are well studied in viral infections, most notably HIV, and strategies have been developed to isolate these highly desirable but rare bnAbs from abundant strain-specific antibodies (27–30). Here, we develop a synthetic discovery strategy to produce bnAbs against 3FTx-L. To enable effective antibody discovery and validation, we assembled a diverse panel of 3FTx-L variants representing toxin antigenic diversity across a collection of medically important snakes. bnAb discovery was performed using a two-part selection strategy, where toxin-specific antibodies were initially selected against a single variant of interest before being split and screened against a small collection of toxins in parallel. Deep-sequencing analysis of these parallel selections then allowed for the high-throughput determination of the small subset of antibodies that recognized an epitope broadly conserved across the different toxin variants. These bnAb candidates were synthesized and characterized to downselect to a lead candidate that was then affinity matured and structurally characterized to elucidate the mechanism of breadth across the diverse 3FTx antigens. Last, in vivo protection against purified native 3FTx-L and crude venom from distinct Asian and African snakes was assessed. This work demonstrates an effective strategy for identifying cross-reactive antibodies over a collection of antigens and provides a template for finding bnAbs that target additional venom toxin classes.

## Results

### Anti-3FTx bnAbs were isolated from a synthetic human Fab library

**Fig. 1:**
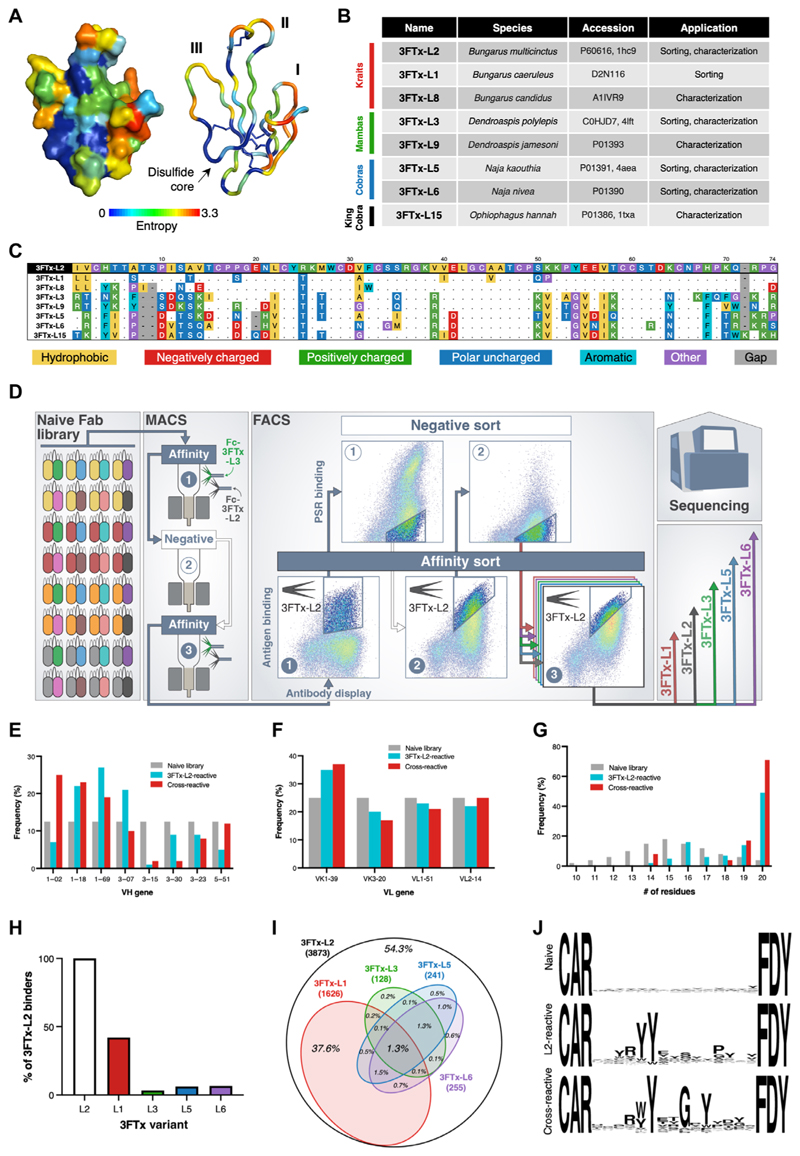
Cross-reactive anti–3FTx-L mAbs were isolated from a naive library. (**A**) Structure of 3FTx-L2 [Protein Data Bank (PDB): 1HC9, (43)] shown as surface (left) and cartoon with the disulfide bonds in the core shown as sticks and labels denoting the locations of loops I, II, and III. The structures are colored by site-specific Shannon entropy values calculated from 149 unique 3FTx-L sequences and mapped onto 1HC9. Red denotes high entropy (sites with substantial antigenic diversity), and blue denotes low entropy (highly conserved positions). (**B**) List of recombinant 3FTx-L variants used in sorting and characterization of mAbs along with corresponding UniProt and PDB accession numbers. (**C**) Sequence alignment of 3FTx-L variants listed in (B) aligned to 3FTx-L2, with dots indicating conserved residues. (**D**) Sorting strategy for selection of cross-reactive Fabs. Fc-fused 3FTx-L2 and 3FTx-L3 were used as baits in MACS. FACS iterated between positive selections with 3FTx-L2 and depletions with polyspecific reagent (PSR) to remove nonspecific antibodies. Final sorts were performed with five different 3FTx-L variants to select for universally enriched cross-reactive clones through deep sequencing. (**E** to **G**) Frequencies of the eight different VH genes (E), four different VL genes (F), and CDRH3 lengths (G) in the naive (unsorted) library, the 3FTx-L2-reactive Fabs, and Fabs that were cross-reactive across all five 3FTx-L variants. (**H**) Percentage of Fab clones present in 3FTx-L2 affinity sort populations also present in the final populations of the four other sort variants as determined by deep sequencing. (**I**) Venn diagram depicting the cross-reactive profile of 3FTx-L2–reactive Fabs. The bold numbers listed in parentheses denote the number of Fabs that bound each 3FTx-L variant, and the italic numbers denote the percentage of Fabs present in each quadrant of the diagram (44). (**J**) Frequency logos constructed with WebLogo (45) for 20-amino acid CDRH3s present in the naive library, the set of 3FTx-L2–reactive Fabs, and the set of cross-reactive Fabs.

Sixteen 3FTx-L variants from a diverse range of medically relevant Asian and African elapid snakes were recombinantly expressed in mammalian cells as candidates for antibody isolation and downstream characterization (table S1). These variants exhibited a broad range of antigenic diversity, with as low as 47% paired sequence identity but with two highly conserved regions: the disulfide core and the second loop (loop II) that is principally responsible for nAChR binding (Fig. 1A) (16). From these 16 candidates, a smaller panel of eight functional variants was constructed to capture overall antigenic diversity and prioritize variants with available published structures (Fig. 1, B and C, and fig. S1). Recombinant 3FTx-L variants used in the antibody isolation campaign were confirmed to bind the human nAChRα1 subunit displayed on the surface of yeast (fig. S2, A and B) and demonstrated antagonism of acetylcholine-induced activation of human muscle–type nAChR in TE671 cells (fig. S2C).

Antibodies against the 3FTx-L variants were isolated from a synthetic human library containing 6 × 10^10^ unique antibodies displayed as Fabs on the surface of *Saccharomyces cerevisiae*. The naive library contained eight variable heavy chain (HC) domains and four variable light chain (LC) domains for a total of 32 variable heavy/light pairs (fig. S3A). The library diversity was introduced into the complementarity determining region 3 of the HC (CDRH3) using trimer phosphoramidite mixtures designed to mimic the distribution of amino acids found in the nontemplated CDRH3 region of naturally occurring human antibodies and included CDRH3 loop lengths from 10 to 20 amino acids (fig. S3, B and C) (31). To efficiently sample the library, we used a dual approach of magnetic-activated cell sorting (MACS) followed by fluorescence-activated cell sorting (FACS) to isolate clones with high affinity and specificity (Fig. 1D and fig. S4). After each selection, the enriched cells were expanded and induced for further rounds of selection. A subset of these cells was reserved for deep sequencing. Two rounds of MACS were performed using 3FTx-L2 and 3FTx-L3 as pooled baits to bulk-enrich anti–3FTx-L reactive Fabs from the naive library. The enriched cells were then subjected to five rounds of FACS: two rounds using decreasing concentrations of 3FTx-L2 to enrich high-affinity clones, interspersed with two rounds of negative selections using soluble cytosolic proteins to deplete poly-reactive clones (fig. S5). After this fourth round of FACS, all cells displayed affinity for 3FTx-L2 and exhibited minimal off-target specificity. To identify broadly cross-reactive Fabs, we split the library into five fractions and screened each against a different 3FTx-L variant in parallel (Fig. 1D and fig. S4). At the conclusion of these selections, cells recovered from each round of FACS were harvested, and the Fab-encoding portions of DNA were deep-sequenced (fig. S6).

In total, we observed 3873 unique Fab sequences that were enriched for binding to the primary antigen, 3FTx-L2 (data file S1). The overall composition of these Fabs was still relatively diverse, with a slight preference for clones that use human HC variable 1 domains (VH1) and minimal LC variable domain (VL) bias (Fig. 1, E and F); however, a notable shift toward 20–amino acid CDRH3 loops was observed (Fig. 1G). The parallel selections with 3FTx-L variants showed a strong correlation between percent identity to 3FTx-L2 and cross-reactivity. 3FTx-L1, with 85% identity with 3FTx-L2, bound 42% of the 3FTx-L2-binding Fabs. The more antigenically distant variants, 3FTx-L3, -L5, and -L6 (50 to 54% identity to L2), cross-reacted with only 3.3, 6.2, and 6.6% of Fabs that bound 3FTx-L2, respectively (Fig. 1H). In total, 52 Fabs (1.3%) were observed to bind all five 3FTx-L variants (Fig. 1I). A majority (71%) of cross-reactive Fabs used a 20–amino acid CDRH3 (Fig. 1G). Examination of this subset showed a predominant [W/Y]YxxGxY motif (Fig. 1J), suggesting that the breadth of reactivity was achieved through a conserved mode of binding.

**Fig. 2:**
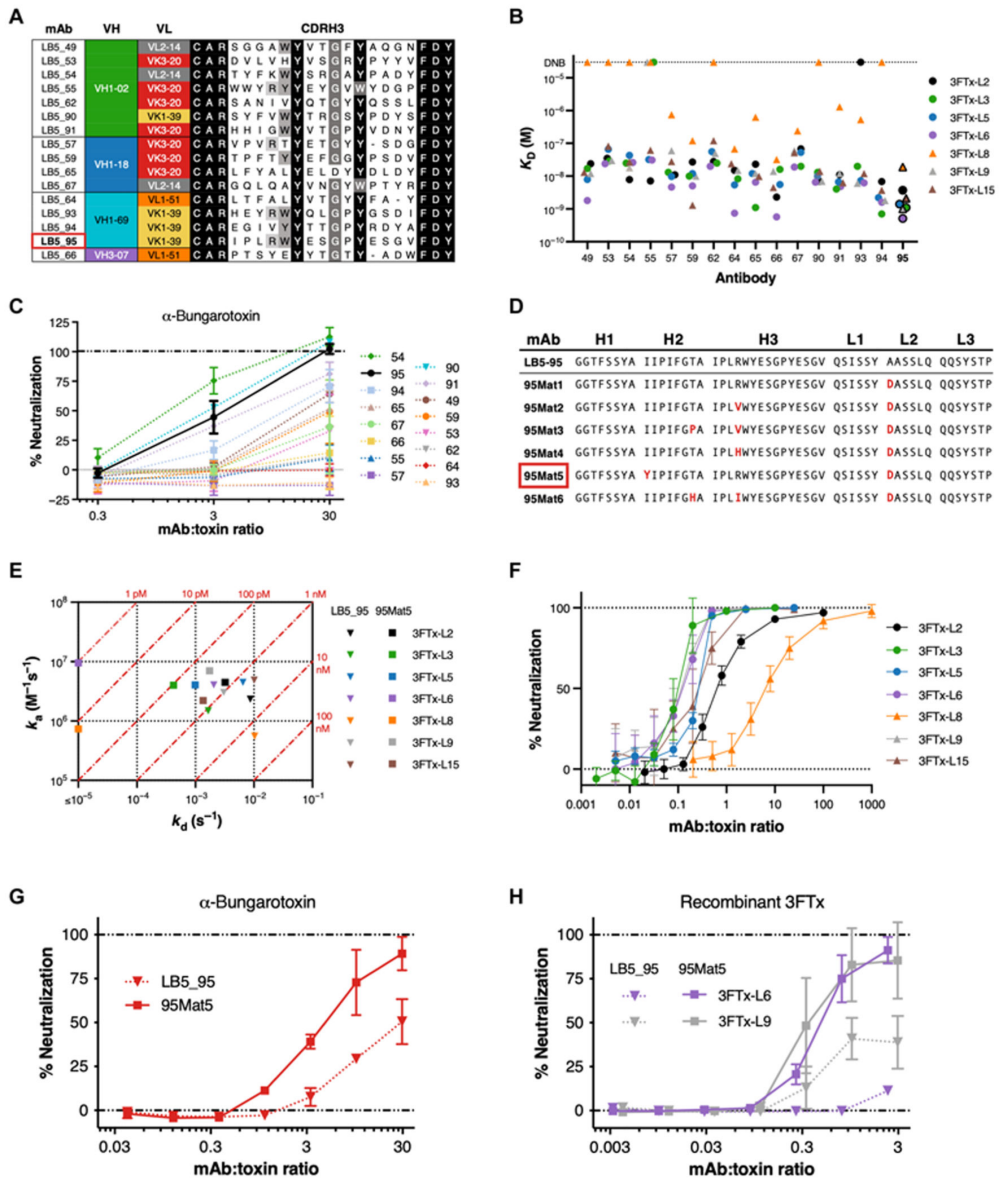
Cross-reactive and affinity-matured antibodies bind and neutralize multiple 3FTx-L variants with high affinity. (**A**) Alignment of CDRH3 sequences for the 16 cross-reactive anti–3FTx-L antibodies isolated and validated from the naive Fab library. LB5_95, the lead candidate, is denoted with a red box. (**B**) Binding affinities across an expanded characterization panel of 3FTx-L variants for each of the cross-reactive antibodies were measured by SPR. 3FTx-L variants from the selections are shown in circles, and additional variants are shown as triangles. (**C**) Functional neutralization of native α-bungarotoxin binding to nAChR by each cross-reactive antibody was evaluated in acetylcholine-induced TE671 cells. Each response was normalized to the maximum (10 μM acetylcholine only) and minimum (10 μM acetylcholine with 30 nM α-bungarotoxin) control responses. Error bars represent the SD from three replicate experiments. (**D**) CDR sequences listed for the six broadly enriched antibodies selected by deep sequencing from the affinity maturation library and the parental antibody (LB5_95). 95Mat5, which was selected for downstream studies, is denoted with a red box. (**E**) Isoaffinity plot of LB5_95 versus affinity-matured 95Mat5 binding to 3FTx-L variants. The measurement of dissociation rate (*k*d) has an instrument limit of 10^−5^ s^−1^, and two points were placed at ≤10^−5^ because they fell below the functional range. (**F**) Blocking of 3FTx-L variant binding to nAChRα1-displaying yeast by 95Mat5 assayed by flow cytometry. The median signal area was normalized to maximum and minimum signals from toxin-only and nAChRα1-only controls to calculate % neutralization. Error bars represent the SD from three to five replicate experiments. (**G** and **H**) Functional neutralization of native α-bungarotoxin (30 nM) (G) and recombinant 3FTx-L6 (380 nM) and 3FTx-L9 (300 nM) (H) binding to nAChR by LB5_95 versus 95Mat5 was evaluated in acetylcholine-induced TE671 cells. Each response was normalized to the control responses as described in (C), with error bars representing the SD of three replicate experiments.

A total of 30 Fabs that were highly enriched in the cross-reactive deep-sequencing datasets were selected to be reformatted as human IgG for functional characterization. All antibodies were tested for binding to 3FTx-L variants and for polyreactive binding to a preparation of detergent-solubilized human embryonic kidney cell proteins. A total of 16 of these antibodies bound all five toxin variants and exhibited little or no off-target reactivity (fig. S7). These antibodies primarily used VH1 HCs and had long (19 to 20 residues) CDRH3s, with most displaying some or all of the conserved [W/Y]YxxGxY motif (Fig. 2A and data file S2). The 16 candidate bnAbs were then tested for binding by surface plasmon resonance (SPR) to an expanded panel of 3FTx-L variants, and all had binding affinities in the 1 to 100 nM range for most of the variants (Fig. 2B). Last, antibodies were also evaluated for neutralization of nAChR antagonism by the native 3FTx-L α-bungarotoxin using TE671 cells (Fig. 2C). Antibody clone LB5_95 was among the most potent neutralizing antibodies, with the highest affinity across all recombinant variants (Fig. 2B), and was therefore chosen as our lead candidate.

During antibody characterization, we noted that although most of the dissociation constants (*K*_D_) for LB5_95 across the 3FTx-L panel were in the 1 to 10 nM range, the dissociation rates (*k*_d_) were relatively fast for an antibody/antigen complex (table S2). We hypothesized that this rapid dissociation rate may be part of the reason that high mAb:toxin ratios were required for complete in vitro neutralization (Fig. 2C). To test this hypothesis, affinity maturation was performed on LB5_95 using our SAMPLER strategy (32), adapted for multistate optimization and with specific emphasis placed on improving the dissociation rate. Initial maturation of the separate HC and LC libraries was performed with both 3FTx-L2 and 3FTx-L3 to maintain cross-reactivity while also decreasing antigen concentrations in successive sorts to select for affinity (fig. S8). The final sorts were performed in parallel with five variants (3FTx-L2, -L3, -L5, -L8, and -L15) at subnanomolar concentrations or with competition sorting to select for variants with the slowest dissociation rates (33). Sequencing analysis of the most broadly enriched clones revealed a highly prevalent aspartic or glutamic acid substituted for alanine in the CDR2 of the LC (CDRL2) (Fig. 2D). Six highly enriched clones were reformatted and expressed as IgG and tested for binding to the panel of 3FTx-L variants by SPR (fig. S9A). Antibody clone 95Mat5 displayed affinity gains across all seven 3FTx-L variants, primarily due to decreased dissociation rates (Fig. 2E) and had a favorable biochemical profile (fig. S9, B to E). 95Mat5 also effectively blocked 3FTx-L variant binding to yeast-displayed nAChRα1 (Fig. 2F). In addition, 95Mat5 demonstrated enhanced functional neutralization of nAChR antagonism in TE671 cells by both α-bungarotoxin and recombinant 3FTx-L variants (Fig. 2, G and H).

### The lead antibody demonstrated broad reactivity across global 3FTx-L variants

**Fig. 3:**
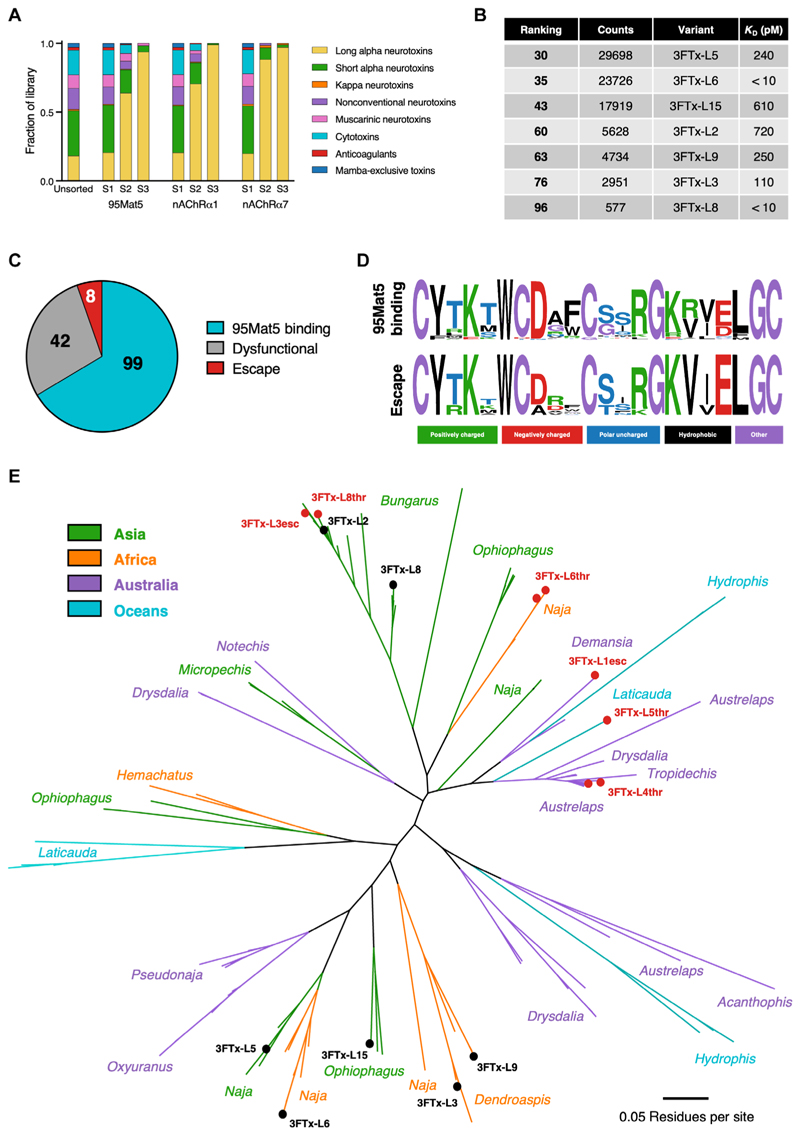
Lead antibody demonstrates broad reactivity across global 3FTx-L variants. (**A**) Composition of the unsorted 3FTx library as compared with the three sequential affinity sorts performed with 95Mat5, nAChRα1, and nAChRα7. 3FTx variants are classified by various known families, and the mamba-exclusive group included fasciculins, FS2 toxins, dendroaspins, and mambalgins. S1, sort 1; S2, sort 2; S3, sort 3. (**B**) List of the deep sequencing ranking and read counts in 95Mat5 sort 3 for the seven 3FTx-L variants used in the characterization panel, along with their respective picomolar dissociation constant (*K*_D_) as measured by SPR. (**C**) Category percentage of library 3FTx-L variants as determined by deep sequencing and ELISA. Dysfunctional variants are nonbinding to both nAChRα1 and nAChRα7, and escape variants are weakly or nonbinding to 95Mat5 but binding to nAChRα1 or nAChRα7. (**D**) Frequency logos constructed with WebLogo (45) for the loop II region of 95Mat5-binding variants versus the escape variants. (**E**) Unrooted tree constructed using the neighbor-joining method with the Jukes-Cantor genetic distance model (Geneious version 2023.1 created by Biomatters) from the alignment of the functional 3FTx-L variants. The genera of the species for the variants in each node are indicated and color-categorized on the basis of the geographic region of the snakes. The node locations of the variants used in antibody characterization are indicated in bold black text, and escape variants are indicated in bold red text using the abbreviated names listed in table S3. A scale bar to represent the degree of genetic change is provided at the lower right.

Having determined that 95Mat5 bound to all seven 3FTx-L variants in our panel with high affinity, we next assessed the true breadth of reactivity across all accessible 3FTx variants from elapid snakes. A yeast display library containing a broad range of 828 publicly available 3FTx variants that included 149 3FTx-L variants was constructed, sorted by three rounds of FACS enrichment with 95Mat5, and analyzed by deep sequencing. Parallel sorts were also performed with human nAChRα1 and nAChRα7 to determine which 3FTx-L variants were nonfunctional (nonbinding to human nAChRα) or mAb-escaping (binding to nAChRα but nonbinding to 95Mat5). The unsorted library contained all 828 3FTx variants at the expected frequencies (Fig. 3A and data file S3), indicating that the construction had been successful. As the selections for binding to 95Mat5 and human nAChRα progressed, a strong enrichment toward 3FTx-L variants was observed (Fig. 3A). A total of 112 3FTx variants were present in the final sort with 95Mat5, which included 105 3FTx-L variants and all variants from our recombinant panel, validating the overall approach (Fig. 3B and data file S3). There was no correlation between the deep sequencing counts and binding affinity of the 3FTx-L variants measured by SPR (fig. S10). To further validate our approach, five 3FTx-L variants present near or below the sequence count threshold were synthesized and tested for 95Mat5-binding by enzyme-linked immunosorbent assay (ELISA). One variant did not express; the other four variants bound 95Mat5, although two were low affinity binders (table S3 and fig. S11A). We also selected six 3FTx-S variants that were present in the final mAb sort for recombinant production and validation. Three 3FTx-S variants failed to express, one bound with low affinity, and the other two did not bind (table S3 and fig. S11A). Collectively, these data indicate that 95Mat5 is highly specific for 3FTx-L variants and does not target other classes of 3FTx.

Next, the sorts for toxin binding to nAChRα1 and nAChRα7 were analyzed to determine the subset of 3FTx-L variants that were functional against the human receptors. Both nAChRα selections enriched almost exclusively for 3FTx-L toxins by the third selection (Fig. 3A and data files S4 and S5). Eleven potential mAb-escaping 3FTx-L variants were expressed and tested for 95Mat5 binding compared with nAChRα binding by ELISA along with the two weakly binding threshold variants (table S3 and fig. S11B). Seven variants showed weak affinity for 95Mat5 with considerably higher affinity for nAChRα7 (fig. S11B), whereas three were dysfunctional, and three were mAbbinding. From the combined sequencing and ELISA data, we determined that 99 of 149 (66%) of the 3FTx-L variants in the library were mAb-binding, 42 of 149 (28%) did not bind either of the human nAChRs, and only 8 (5%) were mAb-escaping while retaining affinity for human nAChRα (Fig. 3C and data file S6). Sequence examination of the mAb-escaping variants revealed that two contained an Asp to Ala mutation in the loop II region of 3FTx-L that may have disrupted mAb-binding (Fig. 3D). A diverse range of 38 elapid species from 16 genera was represented in the 95Mat5-binding set of 3FTx-L variants and spread to geographic regions and alignment nodes that were not encompassed by the characterization panel of 3FTx-L variants (Fig. 3E and data file S6). Collectively, these findings emphasize that the breadth of 3FTx-L binding extends across the clinically relevant elapid snake family.

### 95Mat5 conferred protection against 3FTx-L toxicity in mice

**Fig. 4:**
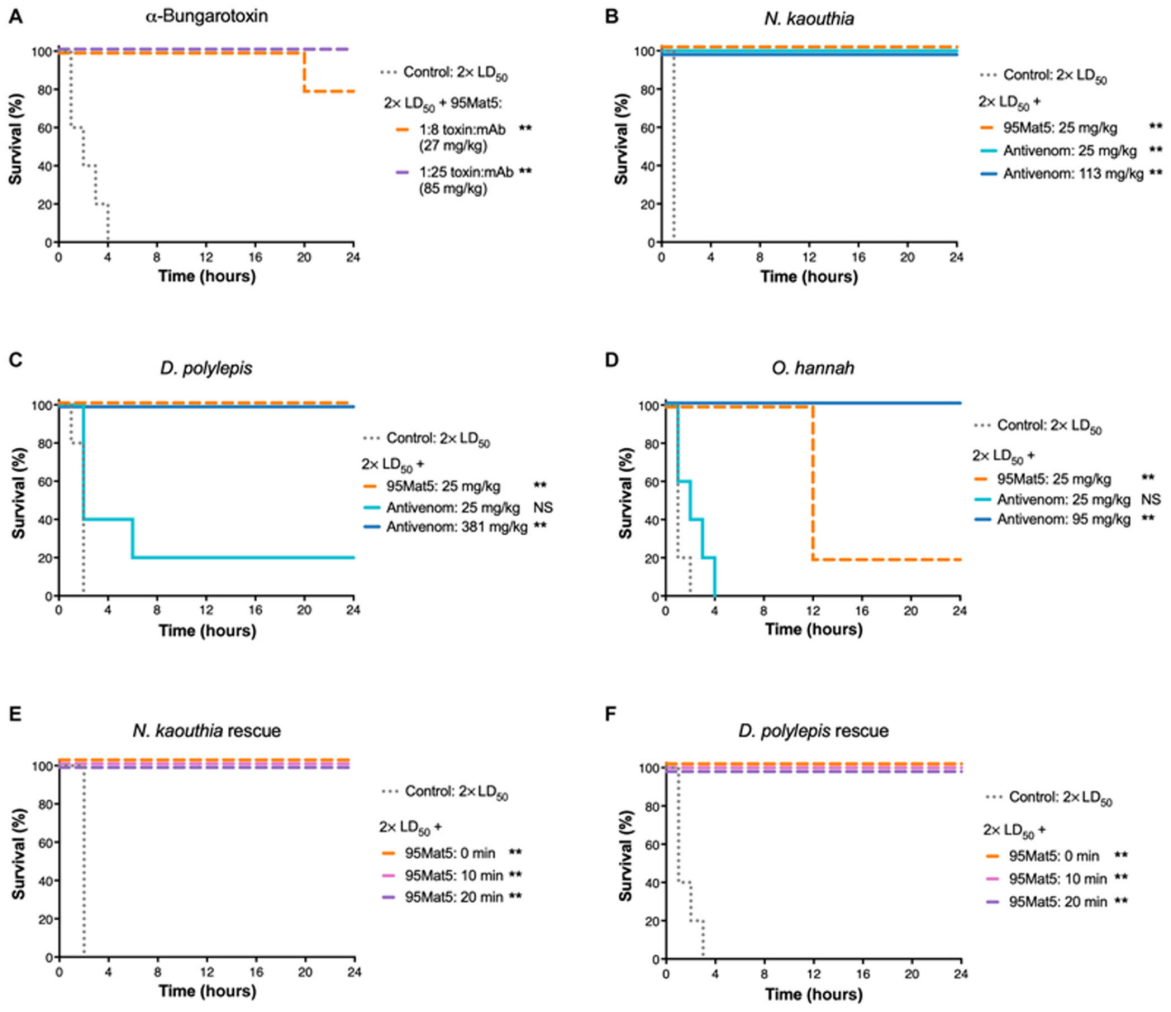
Lead antibody confers protection against 3FTx-L toxicity in mice. (**A**) α-Bungarotoxin (2× LD_50_ dose) was preincubated with 95Mat5 and injected intravenously into groups of experimental animals (*n* = 5) at 1:8 and 1:25 toxin:antibody molar ratios (27 and 85 mg/kg antibody). The control group was injected with purified α-bungarotoxin only. (**B** to **D**) Groups of five mice were challenged with 2× LD_50_ doses of *N. kaouthia* (B), *D. polylepis* (C), or *O. hannah* whole venoms (D). Mice received the mixture preincubated with either no antibody (control), 95Mat5 (25 mg/kg), the manufacturer-recommended dose of commercial antivenom, or an equivalent 25 mg/kg dose of antivenom for direct potency comparison between 95Mat5 and the antivenom. Commercial antivenoms were matched to the appropriate snake. (**E** and **F**) Groups of five mice were injected subcutaneously with 2× LD_50_ doses of whole venoms from *N. kaouthia* (E) or *D. polylepis* (F) before intravenous treatment with 95Mat5 (25 mg/kg) at 0, 10, or 20 min after venom injection. The control groups were injected with venom alone. Significance is noted as follows: **P < 0.005; NS, not significant as compared to control groups.

To determine whether in vitro binding and inhibitory activities translated into preclinical protection against envenoming, we measured the ability of 95Mat5 to protect against the lethal effects of α-bungarotoxin (purified 3FTx-L from *Bungarus multicinctus* venom) and whole snake venoms containing 3FTx-L [*Naja kaouthia, Dendroaspis polylepis* and *Ophiophagus hannah* (34–36)]. First, the median lethal doses (LD_50_) of α-bungarotoxin and crude venoms were determined for administration by both intravenous (IV) and subcutaneous (SC) routes (table S4). Next, groups of five mice received 2× intravenous LD_50_ α-bungarotoxin alone or preincubated with 95Mat5 at molar ratios of 1:8 or 1:25 toxin:antibody. All animals in the control group rapidly exhibited signs of systemic neurotoxicity and succumbed to envenoming within 4 hours, whereas those receiving 95Mat5-preincubated toxin predominantly survived (Fig. 4A). The antibody was able to offer protection against the lethal effects, including rapid reduction of movement, hindlimb paralysis, and loss of righting reflex when compared with the control.

Next, we assessed the cross-neutralizing efficacy of 95Mat5 by preincubating against 2× intravenous LD_50_ challenge doses of *N. kaouthia, D. polylepis*, and *O. hannah* crude venoms. As a positive control and comparator to 95Mat5, we also treated mice with conventional equine-derived commercial antivenoms specific to each of the challenge venoms. These were tested at two concentrations: a high dose equivalent to their marketed neutralizing potency (table S5) and a fixed lower dose of 25 mg/kg for comparison with 95Mat5. Mice intravenously dosed with whole *N. kaouthia* venom succumbed to death within 1 hour, whereas treatment with 95Mat5 or an equivalent antivenom dose provided complete protection for 24 hours (Fig. 4B). 95Mat5 and the higher dose of antivenom protected mice from the lethal effects of *D. polylepis* venom for 24 hours, whereas the dose-matched antivenom yielded only 20% survival (Fig. 4C). The efficacy of 95Mat5 was reduced against *O. hannah* venom (20% survival at 24 hours), although increased animal survival times compared with dose-matched antivenom were observed (Fig. 4D). All experimental animals in this group were active through the first 12 hours of injection, and the surviving individuals did not exhibit any neurotoxic symptoms throughout the observation period, which may suggest that they ultimately succumbed to the toxic effects of other components in the venom.

To better reflect envenomation, we also evaluated the preclinical efficacy of 95Mat5 in a two-step rescue assay where treatment is delivered after venom challenge. We used *N. kaouthia* and *D. polylepis* venoms subcutaneously to evaluate delayed treatment, which consisted of 25 mg/kg of 95Mat5 intravenously at 0, 10, or 20 min after venom dosing. In both groups, control animals died within 3 hours of the venom challenge. In contrast, animals receiving 95Mat5, irrespective of timing, survived the full 24-hour observation period without signs of neurotoxicity (Fig. 4, E and F). Collectively, these data demonstrate that a single mAb (95Mat5) can provide broad preclinical protection against 3FTx-L-containing venoms and exhibits superior dose efficacy than commercial monovalent and polyvalent antivenoms.

### 95Mat5 mimicked the structural interaction of nAChR with 3FTx-L

**Fig. 5:**
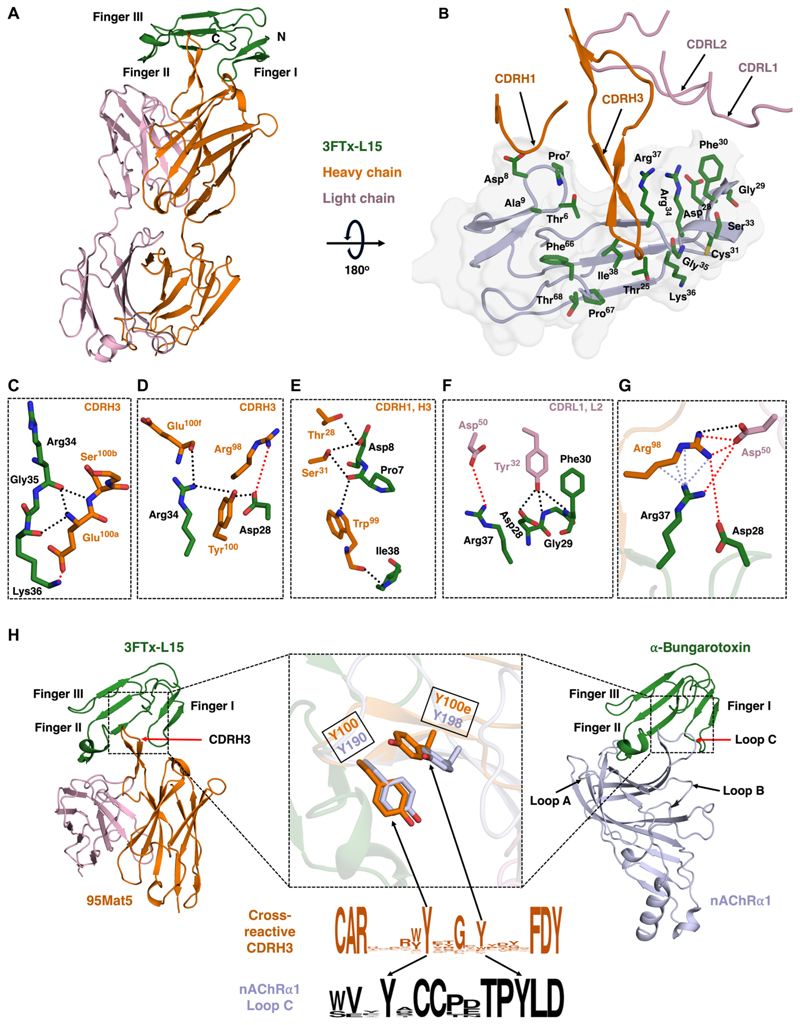
Crystal structure of lead antibody with 3FTx-L15 reveals similarity in toxin recognition by antibody and nAChRα. (**A**) Overall structure of 95Mat5 Fab with 3FTx-L15 in cartoon representation. Orange and pink denote the HC and LC of the Fab, respectively, and 3FTx-L15 is in dark green. (**B**) Interaction interface of 95Mat5 with 3FTx-L15. 3FTXx-L15 epitope residues in dark green sticks. (**C** to **F**) Molecular interactions of 95Mat5 Fab with 3FTx-L15. 95Mat5 Fab CDRH3 with 3FTx-L15 residues (Arg^34^, Gly^35^, and Lys^36^) (C), 95Mat5 Fab CDRH3 with 3FTx-L15 residues (Asp^28^ and Arg^34^) (D), 95Mat5 Fab CDRs H1 and H3 with 3FTx-L15 residues (Pro^7^, Asp^8^, and Ile^38^) (E), and 95Mat5 Fab CDRs L1 and L2 with 3FTx-L15 residues (Asp^28^, Gly^29^, Phe^30^, and Arg^37^) (F). (**G**) Asp^50^ LC of 95Mat5 interacts with and orients Arg^98^ in CDRH3 and is involved in an electrostatic network for toxin recognition. Hydrogen bonds, electrostatic interactions, and van der Waals interactions are represented in black, red, and light blue dashed lines, respectively. (**H**) Comparison of interactions for 95Mat5:3FTx-L15 and nAChRα1:α-bungarotoxin (PDB ID: 2QC1). 3FTx-L15 CDRH3 and nAChRα1 loop C both insert into 3FTx-L in a similar way using two Tyr (Y) residues that are structurally conserved (black box). The 95Mat5 Tyr residues are in orange, and the Tyr residues from nAChRα1 are in lavender. Frequency logos comparing 20–amino acid–long cross-reactive CDRH3 sequences with loop C in nAChRα1 from amphibian, bird, fish, human, lizard, marsupial, rodent, and snake were constructed with WebLogo (45).

To provide a molecular explanation for its broad reactivity, we determined a crystal structure of 95Mat5 Fab in complex with recombinant 3FTx-L15 to 2.9-Å resolution (Fig. 5A and table S6). 95Mat5 targets the central region of 3FTx-L15 (Fig. 5B). The interaction surface is contributed primarily by CDRH3, although CDRs H1, L1, and L2 provide hydrophobic interactions and H bonds (Fig. 5B and table S7). The buried surface area on 3FTx-L15 is 735 Å^2^, with 83 and 17% from the HC and LC, respectively. Specifically, Thr^6^-Ala^9^, Thr^25^-Ile^38^, and Phe^66^-Thr^68^ from 3FTx-L15 are involved, with Asp^28^, Phe^30^, and Arg^34^ interacting with both HC and LC (Fig. 5, B to F).

For the HC, CDRs H1 and H3 of 95Mat5 participate in a polar network, where CDRH3 makes hydrogen bonds and two salt bridges with 3FTx-L15 (Fig. 5, C to E, and table S7) that are crucial for antigen recognition. Polar interactions are formed between (i) Glu^100a^ and Ser^100b^ with 3FTx-L15 Arg^34^ carbonyl oxygen and Lys^36^ amide, (ii) Tyr^100^ hydroxyl and Glu100f carbonyl with 3FTx-L15 Arg^34^ guanidinium and Asp^28^ carboxyl, (iii) CDRH3 Trp^99^ indole NH with 3FTx-L15 Pro^7^ carbonyl oxygen, and (iv) Trp^99^ carbonyl oxygen with 3FTx-L15 Ile^38^ amide (Fig. 5, C to E, and table S7). CDRH1 Thr^28^ and Ser^31^ side chains hydrogen-bond with 3FTx-L15 Asp^8^ carboxyl and Pro^7^ carbonyl oxygen (Fig. 5E and table S7). Hydrophobic interactions are formed between CDRH3 Trp^99^, Tyr^100^, Glu^100a^, and Tyr^100e^ and a pocket lined by 3FTx-L15 Ala^9^, Phe^30^, Arg^34^, Arg^37^, Ile^38^, Phe^66^, Pro^67^, and Thr^68^ (fig. S12 and table S8).

For the LC, 95Mat5 CDRL1 Tyr^32^ hydroxyl hydrogen-bonds with Gly^29^ and Phe^30^ backbone amides as well as Asp^28^ carboxyl (Fig. 5F and table S7). Tyr^32^ and Tyr^92^ interact with Phe^30^ in a hydrophobic pocket on 3FTx-L15 (fig. S13 and table S8). 3FTx-L15 Arg^34^ guanidinium stacks in a cation-π interaction with Tyr^100^ in an aromatic cluster that also involves HC Tyr^100e^, LC Tyr^32^, LC Tyr^92^, and Phe^30^ from its own finger II (fig. S12 and S13, and table S8). This network resembles the interaction of nAChRα1 Tyr^190^ and Tyr^198^ with Arg^36^ and Phe^32^ of 3FTx-L (37). CDRL2 Asp^50^ is involved in electrostatic interactions between two arginines and two aspartic acids. In the Proteins, Interfaces, Structures, and Assemblies (PISA) interaction analysis (see Supplementary Materials), Asp^50^ makes three internal hydrogen bonds/salt bridges with CDRH3 Arg^98^ and a long-range electrostatic interaction with 3FTx-L15 Arg^37^ (Fig. 5, F and G, and tables S7 and S8). 95Mat5 Arg^98^ is also involved in four van der Waals and polar interactions with 3FTx-L15 Arg^37^ and a longer-range electrostatic interaction with 3FTx-L15 Asp^28^ (5.3 Å) (tables S7 and S8). 95Mat5 Asp^50^ therefore plays an important role in the stabilization and orientation of CDRH3 Arg^98^ as well as in longer range electrostatic interactions with 3FTx-L15, thus explaining its strong selection during affinity maturation of LB5_95 (Fig. 2D).

The binding mode of 3FTx-L15 with 95Mat5 is similar to previous structures of α-bungarotoxin with nAChRα1, α7, and α9 (fig. S14) (32–33). 95Mat5 CDRH3 approximates “loop C” of nAChRα1 that inserts between fingers I and II and the C-terminal loop of 3FTx-L toxins (Fig. 5H). The tip of 3FTx-L finger II protrudes into the receptor binding site and is surrounded by loops A, B, and C of nAChRα (Fig. 5H) (38). The antibody and the receptor interact with similar toxin residues (Fig. 5H and table S8). Tyr^100^ and Tyr^100e^ in 95Mat5 align with Tyr^190^ and Tyr^198^ of nAChRα1, respectively. Tyr^100^ is completely conserved among our cross-reactive antibodies (Fig. 5H). Furthermore, 95Mat5 recognizes 3FTx-L15 residues that include Asp^28^, Phe^30^, Arg^34^, Gly^35^, and Lys^36^ on finger II and Pro^67^ on the C terminus (Fig. 5B and table S8). The equivalent α-bungarotoxin residues Asp^30^, Phe^32^, Arg^36^, Gly^37^, Lys^38^, and Pro^69^ also play an important role in nAChR binding (table S8) (39). Thus, our findings suggest that anti-body mimicry of nAChRα1 facilitates broad recognition of 3FTx-L variants by 95Mat5.

## Discussion

Here, we describe the discovery, optimization, and characterization of a broadly neutralizing 3FTx-L antibody that exhibits protective efficacy in mice against lethal challenge by venom from a range of medically relevant snakes. Our project workflow provides a generalizable strategy for finding antibodies that target conserved sites on antigenically variable proteins. The utilization of recombinantly produced toxins allowed for a high degree of control over antigenic variability and bypassed the need to purify the necessary toxins from multiple snake species. The antibody selections from a synthetic library were driven by affinity, overcoming some of the challenges of generating antibodies from animal immunizations using low molecular weight toxins that are often poorly immunogenic. Last, the antibodies recovered from this library are fully human and will therefore lack the typical adverse effects associated with current animal-derived antivenoms. A key advantage of our approach lies in the parallel selections with multiple antigens, allowing for a comprehensive mapping of the cross-reactive profile for all selected antibodies. This strategy allowed for the rapid identification of about 20 bnAbs from the roughly 4000 3FTx-L2-reactive antibodies that were selected from the starting library of 6 × 10^10^ antibodies. Although past work has demonstrated that cross-reactive toxin mAbs are possible using an iterative panning strategy (23), iterative panning is limited in the number of variants that can be included and runs the risk of losing potentially valuable mAbs if an incorrect toxin variant gets included in the selections or if the selection criteria at any step are too stringent.

In vivo protection studies revealed the cross-neutralization potential of 95Mat5, which not only protected against purified α-bungarotoxin (*B. multicinctus*, krait), but also completely neutralized the neurotoxin-rich whole venoms of *N. kaouthia* (monocellate cobra) and *D. polylepis* (black mamba) and prolonged animal survival against *O. hannah* (king cobra). The inability of 95Mat5 to fully protect against *O. hannah* venom could be attributed to this venom’s complexity, being enriched with high proportions of diverse α-neurotoxins, in addition to other 3FTxs and non-3FTx components (36). Structural characterization of 95Mat5:3FTx-L15 complex revealed that the antibody mimics the binding mode of nAChR to 3FTx-L. Receptor mimicry has also been observed in antiviral bnAbs, particularly for HIV and influenza (27, 40), because the receptor recognition site of the viral envelope protein tends to be both highly conserved and critical for viral entry. Given the parallels here, it is perhaps expected that bnAbs against 3FTx-L independently arose with a similar strategy; however, it was notable that this particular solution led to cross-reactivity across all 3FTx-L bnAbs. After screening our naive library, which contained over 60 billion unique antibodies, we recovered only 16 antibodies that bound all variants in our panel. All of the cross-reactive antibodies used a 19– or 20–amino acid CDRH3 loop with most containing a [W/Y]YxxGxY motif, suggesting that all of the mAbs likely use the same overall mode of binding. This result matches previous findings with bnAbs against HIV and influenza, where highly similar antibodies are recovered from multiple donors (41, 42), suggesting a limited number of approaches to achieve broad recognition.

Our study has limitations. Although 95Mat5 demonstrated successful protection of mice against lethal venom challenges from diverse snake species, it should be emphasized that this antibody alone does not constitute the definitive product for universal antivenom development. Because snake venom is a complex cocktail of toxins, it necessitates the inclusion of bnAbs against several additional major venom classes, including 3FTx-S and phospholipases in elapids and metalloproteinases, phospholipases, and serine proteases in viperids (1). Therefore, a final universal antivenom likely would require a minimum of four to five antibodies to effectively cover the additional venom classes, which may include a variety of different mAb formats, including sdAbs or multi-specifics (8) to optimize practical considerations. We also note that our high-throughput cross-reactivity screening against the 3FTx library was a binary analysis of toxin binding to 95Mat5 and nAChRs, whereas protection will likely be dependent on both the binding kinetics of the antibody/toxin and nAChR/toxin, as well as the amount of toxin injected, the dose of antibody administered, the location of toxin administration, and the timing of the events. However, the cross-reactivity analysis in conjunction with the other in vitro and in vivo data presented here demonstrates that 95Mat5 achieves substantial breadth of reactivity across diverse 3FTx-L. The discovery and development of 95Mat5 is an important first step in the development of a monoclonal-based universal antivenom, because it effectively neutralizes one of the most diverse and toxic components of snake venom. Moreover, this research uncovered an adjustable blueprint for generating bnAbs that target antigenically diverse components, providing a valuable framework to develop additional antivenom antibodies.

## Materials and Methods

### Study design

The objective of this study was to find and validate a bnAb against multiple 3FTx-L variants from a synthetic library. Lead antibody candidates were screened and characterized for binding affinities and neutralization potencies. The lead antibody was affinity matured and further validated for broad reactivity and in vivo protection, in addition to determining the crystal structure with its toxin target. For the in vivo studies, the sample size for each experimental group was determined as per WHO’s standard venom-antivenom testing protocols. Experimental animals were manually randomized and assigned to treatment groups without considering any other variable. Experimenters were not blinded to the venom or treatment doses. All the experimental animals were included in the statistical analysis.

### Statistical analysis

The Mantel-Cox log-rank test was performed to estimate the statistical significance of the survival curve comparisons. The Bonferroni method was used to correct for multiple comparisons. Whereas the significance level was set to α = 0.05, the Bonferroni-corrected threshold was α = 0.025 and α = 0.016, where two and three test groups were compared with the venom-alone control, respectively. The comparisons with *P* values higher than the corrected threshold are reported as nonsignificant. All raw, individual-level data for experiments where *n* < 20 are presented in data file S7.

## Supplementary Material

Supplementary Material

## Data Availability

all data needed to evaluate the conclusions in the paper are present in the paper and/or the Supplementary Materials. The coordinates and structure factors for the 95Mat5:3FTx-l15 complex are available in the PdB under accession code 8SXP. Plasmids and reagents will be provided upon request to the corresponding authors after completion of a materials transfer agreement.
