# Supplementary material for: Synthetic development of a broadly neutralizing antibody against snake venom long-chain α-neurotoxins
Source: Sci Transl Med. Author manuscript; Available in PMC 2025 Jun 6. (PMC7617732; doi:10.1126/scitranslmed.adk1867)
Supplement: Supplementary Material [file EMS205028-supplement-Supplementary_Material.zip › scitranslmed.adk1867_sm.pdf]

Science Translational Medicine
AAAS

Supplementary Materials for

Synthetic development of a broadly neutralizing antibody against snake venom long-chain α-neurotoxins

Irene S. Khalek *et al.*

Corresponding author: Kartik Sunagar, ksunagar@iisc.ac.in; Joseph G. Jardine, jardine@scripps.edu

Sci. Transl. Med. **16**, eadk1867 (2024)
DOI: 10.1126/scitranslmed.adk1867

The PDF file includes:

Materials and Methods
Figs. S1 to S14
Tables S1 to S8
Legends for data files S1 to S7
References (*46*–*74*)

Other Supplementary Material for this manuscript includes the following:

Data files S1 to S7
MDAR Reproducibility Checklist

Materials and Methods

Toxin preparation

Long chain three-finger toxin (3FTx-L) variant sequences from various snake species were selected from NCBI databases (**table S1**), codon optimized for mammalian expression, and cloned into a variant of pcDNA3.4 containing an N-terminal signal sequence (VH1-2 leader), a C-terminal Avitag followed by a WELQut cleavage tag, and a rabbit Fc tag by Genscript. A group I phospholipase A2 (PLA_2) from *Naja mossambica* venom (UniProt accession # P00602) was also cloned into the same vector for use in the negative FACS steps. Plasmid DNA was prepped and co-transfected with a bifunctional biotin-[acetylCoA carboxylase] holoenzyme synthetase/DNA-binding transcriptional repressor, bio-5'-AMP-binding (BirA) expression plasmid into Expi293 cells (Thermo Fisher Scientific, cat #A14527) using FectoPRO (Polyplus Transfection). Cells were grown for 5 days with shaking at 240 RPM, 37°C, and 8% CO_2 in Expi293 expression medium (Thermo Fisher Scientific). Approximately 24 hours after transfection, cells were fed 0.4% D-(+)- glucose solution, 3 mM sodium valproic acid solution, and 20 nM d-biotin. Five days post-transfection, the supernatant was harvested from cell cultures, filtered through a 0.45 μm filter, and incubated overnight, rotating at 4°C with rProtein A Sepharose affinity resin (Cytiva). The affinity resin was washed once with 500 mM NaCl in 1X phosphate-buffered saline (PBS) and twice with 1X PBS. For toxin-Fc conjugate purification, elution of the resin was performed with acidic IgG elution buffer (Thermo Fisher Scientific), followed by immediate neutralization to pH 7 using Tris base. The eluent was then concentrated to > 1 mg/ml and exchanged into PBS using a 10 kDa centrifugal filter unit (Millipore Sigma). For cleaved toxin purification, the resin was transferred to 20 mM Tris, 150 mM NaCl, pH 7.4 after washing with PBS. WELQut protease (Thermo Fisher Scientific) was added to the resin and incubated with rotation for 3 hours at 30°C. The supernatant was harvested from the resin and incubated with HisPur nickel-nitrilotriacetic acid (Ni-NTA) resin (Thermo Fisher Scientific) for 30 minutes at room temperature to remove the His-tagged protease, followed by a second incubation with rProtein A resin for 30 minutes to remove any residual Fc tag. The final supernatant was concentrated to >1 mg/ml using a 3 kDa centrifugal filter unit (Millipore Sigma). Toxin-Fc conjugates and cleaved toxins were frozen in aliquots stored at -80°C. Biotinylation of the purified toxins was confirmed using the Pierce biotin quantitation kit (Thermo Fisher Scientific).

For the in vitro neutralization and in vivo protection studies, native α-bungarotoxin purified from *B. multicinctus* venom was purchased from Thermo Fisher Scientific (cat #B1601). The venoms of *N. kaouthia* and *O. hannah* were collected from the wild in India with appropriate permission from the respective forest departments (Kolkata, West Bengal #386/WL/4R-6/2017; 12/02/2018 and Mysore, Karnataka PCCF(WL)/E2/CR-06/2018-19). The *D. polylepis* venom (Zimbabwe) was a kind gift from Premium Serums and Vaccine Pvt. Ltd. (PSVPL).

Antibody library construction and validation

A human antibody library was constructed using the common light chain (LC) strategy *(46)* and displayed as molecular antibody fragments (Fabs) on the surface of *Saccharomyces cerevisiae* in the pYDSI2 Fab display vector, which included a V5 tag for detection of heavy chain (HC) display and a c-Myc tag for detection of LC pairing *(32)*. The library contains 8 human variable heavy (VH) genes (VH1-02, VH1-18, VH1-69, VH3-07, VH3-15, VH3-23, VH3-30 and VH5-51) and 4 human variable light (VL) genes (VK1-39, VK3-20, VL1-51, VL2-14, **fig. S3A**) that were selected based on high-frequency in the human memory compartment, canonical complementary determining region (CDR) structural diversity and favorable developability properties. Library diversity was localized to the CDRH3 using oligonucleotides synthesized with trimer amidite mixtures based on the frequency of amino acids found in human antibody CDRH3 loops, excluding methionines and cysteines *(31)*. The library included CDRH3 lengths between 10 and 20 using a distribution centered on length 15 but skewed to favor longer CDRH3s to avoid oversampling the shorter loops (**fig. S3B**). The VH and VL genes used germline CDR1 and CDR2 loops without any additional diversity, and the CDRL3 loop was fixed based on the most frequently occurring variable/joining rearrangement for each VL gene. To facilitate deep sequencing analysis of selected antibodies, the HCs were designed to have a conserved stretch of nucleotides in the framework 2 region of the VH gene and in the J gene-encoding region just after the CDRH3 (**fig. S6**). Further, each VH gene was codon-optimized in four different ways, and each unique VH nucleotide was paired with one of the 4 fixed LCs. This overall design allows for deep sequencing preparation of a roughly 250 bp stretch of DNA that can be prepared from bulk-sorted cells and encodes the VH gene (identified by the CDRH2 loop sequence), the CDRH3, and the corresponding LC (from the codon optimization of the VH region). DNA for the library was prepared, and cells were transformed using 1,152 replicates of a high-throughput yeast transformation protocol *(31)*. In total, we produced a library with a diversity of $6x10^{10}$ across all VH/VL pairs, estimated by colony-forming unit assay. Upon completion of the library, DNA was harvested from a subset of the expanded cells for deep sequencing analysis of the constructed antibodies. Overall, the CDRH3 length and amino acid distributions closely agree with the targeted distributions (**fig. S3, B and C**).

Magnetic activated cell sorting of the naïve library

Frozen aliquots of the naive library were thawed, and approximately $4x10^{11}$ cells were expanded in 18 L of synthetic-defined yeast medium without uracil (SD-Ura, Sunrise Science) and grown at 30˚C overnight. The following day, approximately $5x10^{11}$ cells were passaged into 18 L of SD-Ura and grown at 30˚C overnight. Cells were then pelleted and resuspended in 18 L of induction media (20 g/L galactose, 1 g/L glucose, 6.7 g/L yeast nitrogen base, 5 g/L bacto-cassamino acids, 38 mM disodium phosphate, 72 mM monosodium phosphate, and 419 µM L-tryptophan), and approximately $4.5x10^{11}$ cells were induced at 18°C for 3 days. For the first round of MACS (MACS1), roughly 10^{12} induced cells were pelleted and washed in PBS, then rotated overnight at 4°C with 20 nM of biotinylated, Fc-conjugated 3FTx-L2 and 3FTx-L3 as antigen baits

in 120 mL PBS with 1% bovine serum albumin, (BSA) to make PBSA. Labeled cells were washed with PBSA in batches of roughly 4.2×10^{10} cells, incubated with 200 µL streptavidin MicroBeads (Miltenyi Biotec) for 20 min at 4°C, and then selected for positive binding using an autoMACS Pro Separator (Miltenyi Biotec). The recovered cells were subsequently grown and induced for negative sorting (MACS2) with streptavidin MicroBeads and anti-biotin MicroBeads in the absence of the antigens (**fig. S4**). For this negative selection step, the streptavidin binders that were enriched from the first round of MACS were discarded. The non-binders were labeled again with the antigens (20 nM Fc-3FTx-L2 and 20 nM Fc-3FTx-L3) for 60 min at 4°C, washed and labeled with anti-biotin MicroBeads, and sorted for positive binding (MACS3). The selected cells were then grown and induced again for FACS.

Fluorescence-activated cell sorting

Yeast cells were iteratively selected for 3FTx binding (affinity sorts), or depleted against polyreactive clones (PSR sorts) by labeling cells with biotinylated polyspecificity reagent *(47, 48)* and selecting the low binding population. After each round of selection, the collected cells were expanded and re-induced prior to the next selection. Subsets of the expanded cells were also frozen for future deep sequencing analysis. During affinity sorting, 1 to 5×10^7 induced yeast cells were incubated for 60 min rotating at 4°C with monomeric biotinylated 3FTx variants in PBSA. For PSR sorting, induced yeast cells were incubated with 20 µg/mL biotinylated HEK-cell soluble cytosolic protein extracts (SCP) *(47)* and 1 µM rabbit Fc-conjugated PLA_2 in PBSA to deplete non-specific and Fc-binding Fab clones. Cells were then washed with PBSA and incubated with streptavidin-allophycocyanin (APC) conjugate (Thermo Fisher Scientific, cat #SA1005), anti-V5 antibody conjugated with Alexa Fluor 405 (AF405), and anti-C-Myc antibody conjugated with fluorescein isothiocyanate (FITC, Immunology Consultants Laboratory, cat #CMYC-45F) in PBSA for 20 min. Yeast cells were then washed once and resuspended in PBSA for sorting on a FACS Melody (BD Biosciences). Paired Fab chains were gated from a plot of AF405 versus FITC signal (HC versus LC), which was subsequently sorted for antigen-binding on a plot of APC versus FITC signal (**fig. S5**). Selected yeast cells were grown in SD-Ura medium shaking overnight at 30°C and induced for consecutive rounds of selection. In the AFF1 and AFF2 sorts, cells were labeled with 100 nM and 20 nM 3FTx-L2, respectively (**fig. S4**). 3FTx-L2 was selected as the primary sort variant due to its complementary native form (alpha-bungarotoxin) being well-characterized and commercially available. In AFF3, the induced library was split into five fractions, each sorted against a different toxin. Cells that received 3FTx-L2 or 3FTx-L1 were sorted at 4 nM, and cells that received 3FTx-L3, 3FTx-L5 and 3FTx-L6 were sorted at 100 nM. AFF4 was also performed using 20 nM 3FTx-L3, 4 nM 3FTx-L5 and 4 nM 3FTx-L6 on the binding populations sorted with those variants during AFF3. Negative PSR sorts were performed between AFF1 & AFF2 and between AFF2 & AFF3 (**fig. S4**). Sorted populations of yeast cells were stored at 4°C in SD-Ura medium until all sorts were completed and ready for sequencing analysis.

Deep sequencing and analysis of selected Fab populations

The archived cells from the FACS selections were passaged in 2 ml SD-Ura medium and grown overnight shaking at 30°C. Yeast cells were then spun down, and the cell pellet was resuspended with 250 µL of buffer P1 with RNAse added (Qiagen) by pipetting up and down. 5 µL of Zymolyase (Zymo Research) was added to digest yeast cell walls and incubated at 37°C for 1 h. Cells were then lysed and neutralized, and DNA was purified according to manufacturer's instructions (Qiagen). A 240 to 290 bp amplicon was prepared by PCR using primers to amplify the HC region of the Fab sequence (forward GGGTAAGACAAGCGCCAGG, forward CAGATGCCAGGAAAGGGCTTG, and reverse CCAACGTACCTTGTCCCCAG) attached to partial Illumina adapters (**fig. S6**) A subsequent round of PCR was performed to attach unique indexing barcodes for each sample library using xGen UDI primers (Integrated DNA Technologies). Once barcoded, all library samples were pooled together, cleaned by column purification (Qiagen) and gel purification, and then measured with a Qubit DNA concentration assay (Thermo Fisher Scientific). The amplicon sample was denatured in 0.1N NaOH, combined with 20% denatured PhiX control library (Illumina) and loaded at 7 pM concentration onto a NovaSeq 6000 System (Illumina) with a paired-end NovaSeq v1.5 500 bp kit.

Paired-end FASTQ files were analyzed using the FastQC package (FastQC v0.11.9) to ensure high sequence quality and low contamination *(49)*. To create one merged contig for each pair of reads, BBMerge (version 38.87) from the BBTools suite was used to join the forward and reverse reads using the default parameters *(50)*. To efficiently quantify the number of identical sequences, the merged reads were clustered using VSEARCH (v2.15.1) *(51)*. Clustering was done using the "cluster_fast" method, and FASTA files were written, including the abundance of each unique sequence in the FASTA header. This step substantially improved the performance of downstream FASTA parsing, as each unique sequence was only analyzed once. Python code (Python 3.7) was written to parse the clustered fasta output, remove primer sequences, identify the base HC and LC, and translate the DNA sequences to amino acid sequences *(31)*. The script then counted the unique CDRH3 sequences separately associated with each HC and LC.

Affinity maturation of LB5_95

Oligo pools for each of the 6 CDRs in LB5_95 were generated with combinatorial point mutations at each site in the CDR using our SAMPLER strategy *(32)* and synthesized by IDT. The mutations were predefined so that the introduction of liabilities such as cysteines, methionines, N-linked glycan motifs, integrin binding sites, and aspartate isomerization sites was avoided. The three CDRH oligo pools were stitched together with the framework regions of VH1-69 to form a heavy chain (HC) library of 5.4×10^6 diversity whereas the three CDRL oligo pools were assembled with the framework regions of VK1-39 to form a light chain (LC) library of 1.4×10^6 diversity. Two additional LC libraries were also generated to combine with the first. One contained combinatorial point mutations in each CDR of VK3-20 and was assembled with the framework regions of VK3-20 with a diversity of 1.6×10^6. The other contained the combinatorial oligo pools for CDRL1 and CDRL2 of VK1-39 along with a pool of 3,500 CDRL3 of lengths varying from 7

to 12 residues retrieved from human naive VK sequences listed on the Observed Antibody Space database *(52)* with a diversity of $3.9x10^7$. The HC library was transformed into yeast with the pYDSI2 Fab display vector containing VK1-39 as the fixed LC. The three LC libraries were transformed into yeast with the same vector containing VH1-69 and the original LB5_95 CDRH3 as the fixed HC. The HC and combined LC libraries were sorted separately with 3FTx-L2 and 3FTx-L3 for the first three affinity sorts (AFF1-AFF3), with a negative PSR sort (PSR1) performed between AFF2 and AFF3 using 20 µg/mL SCP (**fig. S8**). In AFF1, 4 nM and 1 nM of each 3FTx variant were used for the HC and LC libraries, respectively. Both libraries were then sorted with 200 and 40 pM of both 3FTx variants in AFF2, which were combined in PSR1 (**fig. S8**). In AFF3, the separate HC and LC PSR1 libraries were each sorted with 3FTx-L2 (40 pM) and 3FTx-L3 (40 pM). To avoid depleting conditions, the binding of 10^7 cells at low antigen concentrations was performed in larger volumes (5 ml, 30 ml, and 140 ml for 1 nM, 200 pM, and 40 pM, respectively).

After the first rounds of sorting with the separate HC and LC libraries, the VH and VK DNA from the enriched Fab clones were extracted and assembled to form a combined HC/LC library (**fig. S8**). This library was transformed into yeast with the dual vector for subsequent rounds of sorting. Affinity sorts 4 to 6 were performed with the combined HC and LC library, with a negative PSR sort (PSR2) between AFF4 and AFF5 (**fig. S8**). In AFF4, the combined library was sorted with 1 nM, 200 pM, and 40 pM of both 3FTx-L2 and 3FTx-L3. These populations were combined together during PSR2 and sorted with 20 µg/mL SCP. In AFF5, the library was split again and sorted with 3FTx-L2 (40 pM), 3FTx-L3 (200 pM), 3FTx-L5 (40 pM), 3FTx-L8 (1 nM), and 3FTx-L15 (40 pM). An additional affinity sort (AFF6) was performed for the populations sorted with 3FTx-L2, 3FTx-L8, and 3FTx-L15 (**fig. S8**). Dissociation conditions (competition sorting) were used for 3FTx-L2 and 3FTx-L15 during AFF6. Briefly, cells were incubated with 10 nM of biotinylated toxin, washed in PBSA, and then incubated overnight with 10 nM of non-biotinylated toxin at 4°C. The following day, cells were washed, incubated with secondaries, and sorted to select antibody clones that had retained binding to the biotinylated toxin overnight. For sequencing of the affinity maturation library, a 570 bp amplicon containing all six CDR sites was prepared from a circularized PCR product that ligated the CDR3 end of the HC to the CDR3 end of the LC. The amplicon was processed as described above and sequenced on a MiSeq System (Illumina) with a paired-end MiSeq v3 600 bp kit.

IgG expression, purification, screening, and characterization

The selected antibodies were reformatted as IgG1 and synthesized by GenScript into a mammalian expression vector containing both the HC and LC separated by a P2A self-cleaving motif. Antibody encoding plasmids were transfected into Expi293 cells, purified using Protein A magnetic beads (Thermo Fisher Scientific), and eluted with acidic IgG elution buffer. An ELISA was used to screen for the cross-reactive binding of the antibodies against 3FTx-L variants. Briefly, polystyrene high-bind microplates (Corning, cat # 3690) were coated with anti-rabbit IgG Fc antibody (Sigma, cat # SAB3700849) overnight at 4°C, washed 3X with PBS containing 0.05% Tween-20 (PBST), blocked with PBS containing 3% BSA, incubated with recombinant rabbit Fc-

tagged 3FTx-L for 1 hour at room temperature, and washed again 3X with PBST. Plates were then incubated with serial dilutions of the eluted antibodies for 1 hour, washed 3X with PBST, and incubated for 1 hour with alkaline phosphatase-conjugated anti-human IgG Fc-specific antibody (Jackson ImmunoResearch, cat # 109-055-098), and washed a final 3X with PBST before incubating with phosphatase substrate (Sigma cat # S0942) and reading absorbance at 405 nm. Antibodies that showed positive binding to 3FTx-L2 were then tested for non-specific binding on ELISA plates coated with Chinese hamster ovary cell soluble membrane protein extract (CHO-SMP). Antibodies that did not bind CHO-SMP were then screened for binding against additional 3FTx variants (3FTx-L1, 3FTx-L3, 3FTx-L5, and 3FTx-L6) by ELISA.

Sixteen of the 30 antibodies selected for cross-reactivity by deep sequencing screened positive for cross-reactive binding to all 5 toxin variants (**Fig. 2A**). These antibodies were recloned into mammalian expression vectors containing only the HC, co-transfected with the corresponding LC using a 1:2.5 ratio of HC:LC DNA and purified from Expi293 cells with rProtein A Sepharose affinity resin (Cytiva) and elution with acidic buffer. The purity and monomeric content of the antibodies were analyzed using SDS-PAGE and analytical size exclusion chromatography on a 1260 Infinity II preparative LC system (Agilent) using a TSKgel SuperSW mAb HR HPLC column (Tosoh Bioscience). Non-specific binding of these antibodies was further evaluated with the HEp-2 assay (Hemagen) using methods provided by the manufacturer and compared with reactive (bococizumab) and non-reactive (adalimumab) antibody controls.

Six cross-reactive antibody candidates were selected from the affinity maturation library for synthesis and testing. The antibodies were expressed as IgG1 using a 1:2.5 ratio of HC:LC DNA and purified as previously described. The antibodies were first tested for non-specific binding on ELISA plates coated with CHO-SMP, single-stranded DNA, or insulin as described above (**fig. S9B**) and subsequently tested for self-association using affinity-capture self-interaction nanoparticle spectroscopy (AC-SINS, **fig. S9C**). Briefly, 20 nm gold nanoparticles (Ted Pella) were coated for 1 hr at room temperature with a 4:1 mixture of anti-human IgG Fc-specific capture antibodies (Jackson ImmunoResearch, cat #109-005-098) and polyclonal goat IgG non-capture antibodies (Jackson ImmunoResearch, cat #005-000-003) that had been buffer-exchanged into 20 mM sodium acetate, pH 4.3, and normalized to a concentration of 0.4 mg/ml. The coated nanoparticles were then blocked with 0.1 μM thiolated poly(ethylene glycol) for 1 hr and then concentrated and buffer-exchanged into PBS. The maturation antibody candidates were then incubated with the concentrated nanoparticles at a 9:2 volume ratio for 1 hr with a final Ab concentration of 41 μg/ml. The average plasmon wavelength was calculated as the wavelength causing maximum absorption at 510 to 570 nm for each antibody sample, and the plasmon wavelength from a blank control was subtracted to determine the wavelength shift caused by antibody self-association. A non-self-interacting antibody (adalimumab) and a self-interacting antibody (bococizumab) were utilized as controls for comparison. The antibodies were additionally characterized by analytical SEC and HEp-2 assay as previously described (**fig. S9, D and E**). The lead maturation candidate (95Mat5) was also expressed and purified from Expi293 cells in a His-tagged Fab format (VH conjugated to constant CH1-only) for use in the structural

studies. Briefly, harvested supernatant was incubated with HisPur Ni-NTA resin (Thermo Fisher Scientific) and eluted using 500 mM imidazole, then run over SEC using a HiLoad 16/600 Superdex 200 pg column (Cytiva) on an AKTA pure protein purification system (Cytiva).

Surface plasmon resonance

SPR measurements were carried out on a Biacore 8K instrument at 25°C. All experiments were performed with a flow rate of 30 μL/min in a mobile phase of HBS-EP+ (0.01 M HEPES (pH 7.6), 0.15 M NaCl, 3 mM EDTA, 0.0005% (v/v) Surfactant P20). Anti-Human IgG (Fc-specific) antibody (Cytiva, cat # BR100839) was immobilized to a density of about 3000 to 5000 resonance units (RU) by standard NHS/EDc coupling to a Series S CM-3 (Cytiva) sensor chip. A reference surface was generated through the same method.

For conventional kinetic/dose-response experiments, listed antibodies were captured to 50 to 100 RU by Fc-capture on the active flow cell prior to analyte injection. A concentration series of 3FTx-L variants or clinically relevant antigens were injected across the antibody and control surface for 2 min, followed by a 20 min dissociation phase using a multi-cycle method. Regeneration of the surface in between injections of analyte was achieved with two 2 min injections of 3 M $MgCl_2$. Kinetic analysis of each reference subtracted injection series was performed using the BIAEvaluation software (Cytiva). All sensorgram series were fit to a 1:1 (Langmuir) binding model of interaction.

Flow cytometry nAChR blocking assay

The construct for the nAChRα1 subunit of the nicotinic acetylcholine receptor (nAChR) was based on a sequence used previously *(53)* and displayed on the surface of *S. cerevisiae* in the pYDSI2u_SiDi1 vector *(31)*. Biotinylated, cleaved 3FTx-L variants were pre-coupled with 95Mat5 antibody in various ratios for 1 hour at 4°C prior to incubating with induced yeast cells displaying nAChRα1 for an additional hour in volumes avoiding depletion conditions. Cells were then washed with PBSA and coupled with streptavidin-APC and anti-V5-AF405 for 20 min prior to flow cytometry on a ZE5 Cell Analyzer (BioRad). Non-linear curve-fitting to determine the half-maximal effective concentration (EC_{50}) for each 3FTx-L variant binding to the displayed nAChRα1 subunit was performed in Prism 10 (GraphPad) using a one-site competitive binding model (**table S1** and **fig. S2**), and the EC_{50} concentration of each variant was applied in the blocking assay.

TE671 cell-based assay of nAChR antagonism

A fluorescence assay measuring the activation of fetal (γ-subunit-containing) muscle-type nAChRs natively expressed in the TE671 cell line (RRID: CVCL_1756) using a membrane potential dye (FLIPR membrane potential dye blue, Explorer Kit, R8042, Molecular Devices) was employed as described in Patel *et al. (54)*. All further reagents in this section were acquired from Gibco, Thermo Fisher Scientific unless stated otherwise. TE671 cells were cultured in a culture medium consisting of Dulbecco's Modified Eagle Medium with GlutaMAX supplement, further

supplemented with 10% fetal bovine serum and 1% penicillin-streptomycin solution and incubated at 37 °C/5% CO_2 until ~90% confluence was reached. Cells were dislodged from cell culture flasks with TrypLE express enzyme, and the resulting suspension was added to a 10 mL culture medium, followed by centrifugation for 5 minutes at 300 x g. The supernatant was removed, the pellet was resuspended in a 5 mL culture medium and then counted using an automated cell counter (Luna II, Logos Biosystems), and medium was added to reach a count of (5 to $6x10^4$ cells/100 µL). 100 µL of cell suspension was then seeded into a clear-bottom, black-walled 96-well plate then incubated overnight at 37 °C/5% CO_2.

One vial of membrane potential dye was reconstituted in 36 mL assay buffer, which consisted of Hank's Balanced Salt Solution (HBSS, created from 10x solution as per manufacturer's instructions) supplemented with 20 mM HEPES and 0.5 µM atropine (A0132, Sigma-Aldrich) and sterile filtered. Assay buffer was also used to create all solutions containing toxin, antibody, or acetylcholine. The culture medium was removed and replaced with 50 µL of dye solution and incubated for 30 min at 37 °C/5% CO_2. For antibody neutralization experiments, solutions of toxin, antibody, toxin + antibody or assay buffer alone were concurrently incubated in a clear v-bottom 96-well plate ('reagent plate') (651201, Greiner Bio One). Reagent plates were not incubated for toxin half-maximal inhibitory (IC_{50}) experiments. After incubation, 50 µL solution from the reagent plate was transferred to the cell plate, and the cell plate was incubated for a further 15 min at 37 °C/5% CO_2. Following incubation, the cell plate was acclimatized at room temperature for 15 min then recorded using a FlexStation 3 multi-mode microplate reader controlled by SoftMax Pro 7.1 software (Molecular Devices). Excitation, cut-off, and emission wavelengths were set at 530, 550, and 565 nm, respectively, and recordings were carried out at room temperature with a read time of 214 s and an interval time of 2 s. A compound transfer of 50 µL of either 30 µM acetylcholine solution (to give 10 µM final concentration) or assay buffer alone from a separate clear v-bottom 96-well plate loaded into the reader was set after 20 s baseline recording to induce nAChR activation.

Fluorescent responses were measured by the software in relative fluorescence units (RFUs), and the response for each well was determined by calculating the baseline (mean of the first 20 s of recording) and subtracting this from the maximum response from the remainder of the recording. For experiments with toxins only, 10 µM acetylcholine and assay buffer alone were used as controls, with all data points normalized to the mean of the acetylcholine-only responses. For experiments with antibodies and toxins, toxin-only and antibody-only controls were also included, with all data points normalized to the mean of the acetylcholine-only and toxin-only controls. This was achieved by subtracting the toxin only control from the antibody-plus-toxin and acetylcholine-only responses before normalizing to the acetylcholine response. To ensure a maximal signal window, varying concentrations of antibody were incubated with toxin concentrations that gave final concentrations of 30 nM α-bungarotoxin (as used in Patel *et al. (54)*) and 380 nM 3FTx-L6 or 300 nM 3FTx-L9 (calculated after IC_{50} analysis of toxin in **fig. S2**). All experiments had 2 to 4 replicates per plate, and experiments were repeated on 3 separate days using different cell passage numbers. The mean of the replicates for each plate was calculated and

combined with the means from the 2 other plates to give n=3. The means of these combined values were then calculated and plotted as the mean ± SD. Prism 9 (GraphPad) was used for all graph plotting and application of non-linear regression equations to determine IC_{50} values.

Screening and selection of antibody-binding toxins

A yeast display library containing 828 3FTx variants was constructed and synthesized as an oligo pool by Twist Bioscience. These variants were picked from NCBI databases and transcriptome sequencing *(55–58)* to cover a broader range of snake species and encompass other 3FTx families beyond α-neurotoxins, including muscarinic neurotoxins, cytotoxins, and anticoagulants, while also containing 149 3FTx-L and 272 3FTx-S variants (**data file S3**). To enhance the coverage of the library, the 3FTx oligo pool contained three different codon versions of each protein sequence (2,484 total oligos). The 3FTx library was displayed on the surface of *S. cerevisiae* in the pYDSI2u_SiDi1 vector. A series of 3 affinity sorts were performed using 95Mat5 antibody at a concentration of 20 nM on a FACS Melody (BD Biosciences). All binding clones gated above the background display signal were isolated in each sort. Parallel sorts were performed with 20 nM of purified nAChRα7 and mammalian cell supernatant containing nAChRα1. The constructs for nAChRα1 and α7 were based on sequences used previously *(53, 59)* and were conjugated to either a human Fc tag or both His and human Fc tags, respectively. In the final affinity sort with the antibody, populations of high affinity, moderate affinity, and low affinity clones were isolated in addition to the entire binding population. Yeast cells were incubated with antibody in PBSA for 1 hour at 4°C prior to washing and incubation with anti-V5-AF405 conjugate and anti-human IgG Fc-phycoerythrin (PE) conjugate (Southern Biotech cat #9040-09) or anti-His-APC conjugate (Miltenyi Biotec 130-119-782) for 20 min to select for cells displaying toxin and binding to antibody or nAChRα. Isolated yeast cells were grown in SD-Ura medium shaking overnight at 30°C and induced for consecutive rounds of selection. Reserved cells from each sort were prepared for deep sequencing of the toxin-encoding plasmid region using a 330 to 360 bp amplicon attached to partial Illumina adapters and methods described above. Deep sequencing was performed using a NextSeq System (Illumina) with the paired-end NextSeq 1000/2000 P1 600 bp kit.

To analyze the results, a composite reference of the 2,484 3FTx nucleotide sequences was constructed in FASTA format and indexed using Burrows-Wheeler Transform *(60)*. Forward and reverse reads were merged using BBMerge (version 38.87) from the BBTools suite with default parameters *(50)*. Merged reads were aligned to the reference and quantified using SAMtools *(61)* without removing duplicates. Variants enriched and expressed through at least two of the codon versions in the final sort were considered to be antibody or nAChR-binding. An enrichment threshold of 10 total counts from the top two codon versions was applied in all sorts performed with nAChRα and the first two sorts with 95Mat5. Variants were filtered from those that were present in the previous sort. In the third sort with 95Mat5, the sum of the counts in all four populations (all binders, high affinity, moderate affinity, and low affinity) was used to determine binding by applying a threshold of 100 total counts. Five 3FTx-L variants present near or slightly

below the sequencing data threshold, and six 3FTx-S variants present in the final sort with 95Mat5 were selected for synthesis and binding validation by ELISA (**table S3 and fig. S11A**). An additional eleven 3FTx-L variants that were present in the final nAChRα sorts but not the final antibody sort were also synthesized and tested for binding with 95Mat5 compared with nAChRα7 by ELISA (**table S3 and fig. S11B**). Recombinant expression and ELISA were performed using the rabbit Fc-conjugated toxin construct and methods previously described.

Shannon entropy calculations and 3FTx-L2-binding Fab analysis

The Shannon entropy used in **Fig. 1A** was calculated for each position in the alignment of 3FTx-L library variants using the equation $H(x) = -\sum_{i=1}^{n} p(x_i)\, log_2 p(x_i)$ where $p(x_i)$ is the frequency of each amino acid residue or gap ($n = 21$) occurring throughout the collection of 149 variants. The 3FTx-L2-binding Fab clones quantified in **Fig. 1, E to I** were filtered from the deep sequencing data of the naive library sorted with 3FTx-L2 based on a total of 100 or more counts across the combination of AFF2, PSR2, and AFF3 sorts. PCR sequencing artifacts and any sequence with a stop codon were removed. Cross-reactive Fab clones were filtered out of the 3,873 Fabs that bound 3FTx-L2 based on their presence in all four of the final affinity sorts for 3FTx-L1, L3, L5 and L6 above a threshold of 10 counts (**data file S1**).

Animal ethics and biosafety clearances

Approvals to perform in vivo neutralization assays in the murine model of envenoming were obtained from the Institutional Animal Ethics Committee, Indian Institute of Science, Bangalore (CAF/Ethics/904/2022 and CAF/Ethics/947/2023). Experimental protocols were designed following the guidelines issued by the Committee for Control and Supervision of Experiments on Animals (CCSEA), Government of India. The Institutional Biosafety Committee (IBSC) permission was also obtained as these experiments involved the use of snake venoms and recombinant proteins. Healthy CD-1 male mice were purchased from Hylasco Biotechnology (India) Pvt. Ltd and housed at the Central Animal Facility, IISc. The animals were quarantined for seven days prior to experimentation. Three- to four-week old mice, weighing between 18 and 20 g, were randomly allocated and maintained in groups of 5 per cage with food and water ad libitum. The cages were provided with appropriate bedding material and maintained at 18 to 24 °C, 60 to 65% relative humidity and a 12:12 day-night cycle. The sample size for each experimental group was determined as per WHO's standard venom-antivenom testing protocols. Experimental animals were manually randomized and assigned to treatment groups without considering any other variable. Experimenters were not blinded to the venom or treatment doses. All the experimental animals were included in the statistical analysis. Analgesics were not administered to avoid interference with the symptoms of neurotoxic envenoming (such as respiratory depression).

Determination of median lethal dose

The intravenous and subcutaneous median lethal doses (LD_{50}) of α-bungarotoxin and crude venoms from *N. kaouthia, D. polylepis,* and *O. hannah* were determined in mice (male CD-1 mice; 18 to 22 g) using the WHO-recommended approach *(62)* (**table S4**). Five concentrations of toxin or venom were diluted in PBS to a volume of 200 μl and then intravenously (caudal vein) or

subcutaneously injected into groups of five mice. The mortality rate was recorded every one hour for a total of 24 hours post-venom injection, and the LD_{50} was estimated using Probit statistics *(63)*.

Preincubation neutralization experiments

The in vivo neutralizing efficacy of 95Mat5 against α-bungarotoxin and *N. kaouthia, D. polylepis* and *O. hannah* venoms was evaluated in mice (male CD-1 mice; 18 to 22 g; n=5 per group) using a WHO-recommended preincubation and intravenous co-administration approach *(62)*. A 2x LD_{50} dose of α-bungarotoxin (22 µg/mouse in PBS) was incubated with 95Mat5 antibody in toxin:antibody molar ratios of 1:8 and 1:25 (27 and 85 mg/kg antibody) at 37 °C for 30 min. The preincubated mixture (200 µl volume) was then intravenously injected into the caudal vein of a group of five mice, and the animals were continuously monitored over a 24-hour period. The time taken for the appearance of pathophysiological symptoms (e.g., hindlimb paralysis, loss of righting reflex, etc.) and death was noted. Kaplan–Meier plots were generated to depict the percentage survival over time and Mantel-Cox Log-rank test was performed to estimate the statistical significance. Similar experiments were conducted with 2x LD_{50} doses of each crude venom preincubated with 25 mg/kg of 95Mat5 antibody. The efficacy of 95MatAb was evaluated in comparison with the conventional monovalent antivenoms marketed by Queen Saovabha Memorial Institute (QSMI), Thailand, for treating *N. kaouthia* and *O. hannah* envenoming, and African polyvalent antivenom marketed by PSVPL for treating *D. polylepis* bites. The contents of the commercial antivenom vial were weighed and reconstituted in 10 ml sterile water provided by the manufacturer. These conventional antivenoms were tested both at 25 mg/kg and at doses equivalent to their marketed neutralization potencies (**table S5**). In all the aforementioned experiments, a group of five mice injected with α-bungarotoxin or respective crude venom alone served as a positive control.

Rescue experiments

The ability of 95Mat5 to rescue experimental animals injected with crude snake venom was assessed using a two-step rescue strategy to better reflect a clinical envenoming scenario *(64)*. Since 95Mat5 exhibited reduced efficacy against *O. hannah* venom in the preincubation studies, only *N. kaouthia* and *D. polylepis* venoms were selected for rescue experiments. In these experiments, a 2x LD_{50} dose of each venom (27.52 and 14.34 µg/mouse for *N. kaouthia* and *D. polylepis,* respectively), diluted in PBS to 100 µl, was subcutaneously injected into groups of five mice, followed by the intravenous administration of 95Mat5 (25 mg/kg in 100 µl PBS) at 0, 10, or 20 minutes post-venom injection. For comparison, the conventional monovalent antivenom against *N. kaouthia* (QSMI) was also estimated at the same 25 mg/kg dose in PBS and injection time points (0, 10, or 20 minutes). Mice were observed over a 24-hour period, and the percentage survival over time was plotted as Kaplan–Meier curves. The Mantel-Cox Log-rank test was performed to estimate the statistical significance.

Structural studies

3FTx-L15 was mixed with 95Mat5 Fab at a 1:1.3 molar ratio of toxin:Fab. The mixture was incubated overnight at 4ºC before further purification by gel filtration (Superdex 200 column) to remove uncomplexed toxin or Fab. The peak corresponding to the complex was pooled and adjusted to 12 mg/mL in 20 mM Tris pH 8.0, and 150 mM NaCl buffer. The complex between toxin and Fab was confirmed by SDS-PAGE and used immediately for crystallization trials. The antibody-antigen complex was screened for crystallization using the 384 conditions of the Joint Center for Structural Genomics (JCSG) Core Suite (Qiagen) using the sitting drop vapor diffusion method on our automated CrystalMation robotic system (Rigaku) at The Scripps Research Institute. Within 3 to 7 days, diffraction-quality crystals were obtained using 100 mM sodium citrate pH 3.8 and 26% PEG6000 as precipitant at 20°C.

Crystals were cryoprotected with 15 to 25% ethylene glycol and were flash-cooled and stored in liquid nitrogen until data collection. Data were collected from a crystal cryocooled to 100K using a Rigaku MicroMax-007 generator at 1.5418 Å wavelength with a Mar345dtb area detector. Diffraction data were processed with HKL-2000 *(65)*. The initial models for 95Mat5 Fab were generated by Repertoire Builder (https://sysimm.ifrec.osaka-u.ac.jp/rep_builder/) *(66)*. The model template for 3FTx-L15 was derived from α-bungarotoxin V31 (PDB: 1HC9). Initial phases were determined by molecular replacement using Phaser *(67)*. Refinement was carried out in Refmac *(68)* and Phenix *(69)*, alternating with manual rebuilding and adjustment in COOT *(70)*. Detailed data collection and refinement statistics are summarized in Table S6. Structure validation was performed with The Protein Data Bank validation server and MolProbity *(71)*. Before Protein Data Bank (PDB) deposition, PDB-REDO *(72)* was performed to complete the structure refinement. For the Fabs, the residues were renumbered according to Kabat nomenclature *(73)*. Epitope, paratope residues, buried molecular surface area, as well as their interactions, were identified by accessing PISA at the European Bioinformatics Institute (www.ebi.ac.uk/pdbe/pisa/) *(74)*. Structure figures were generated by MacPyMol (DeLano Scientific LLC).

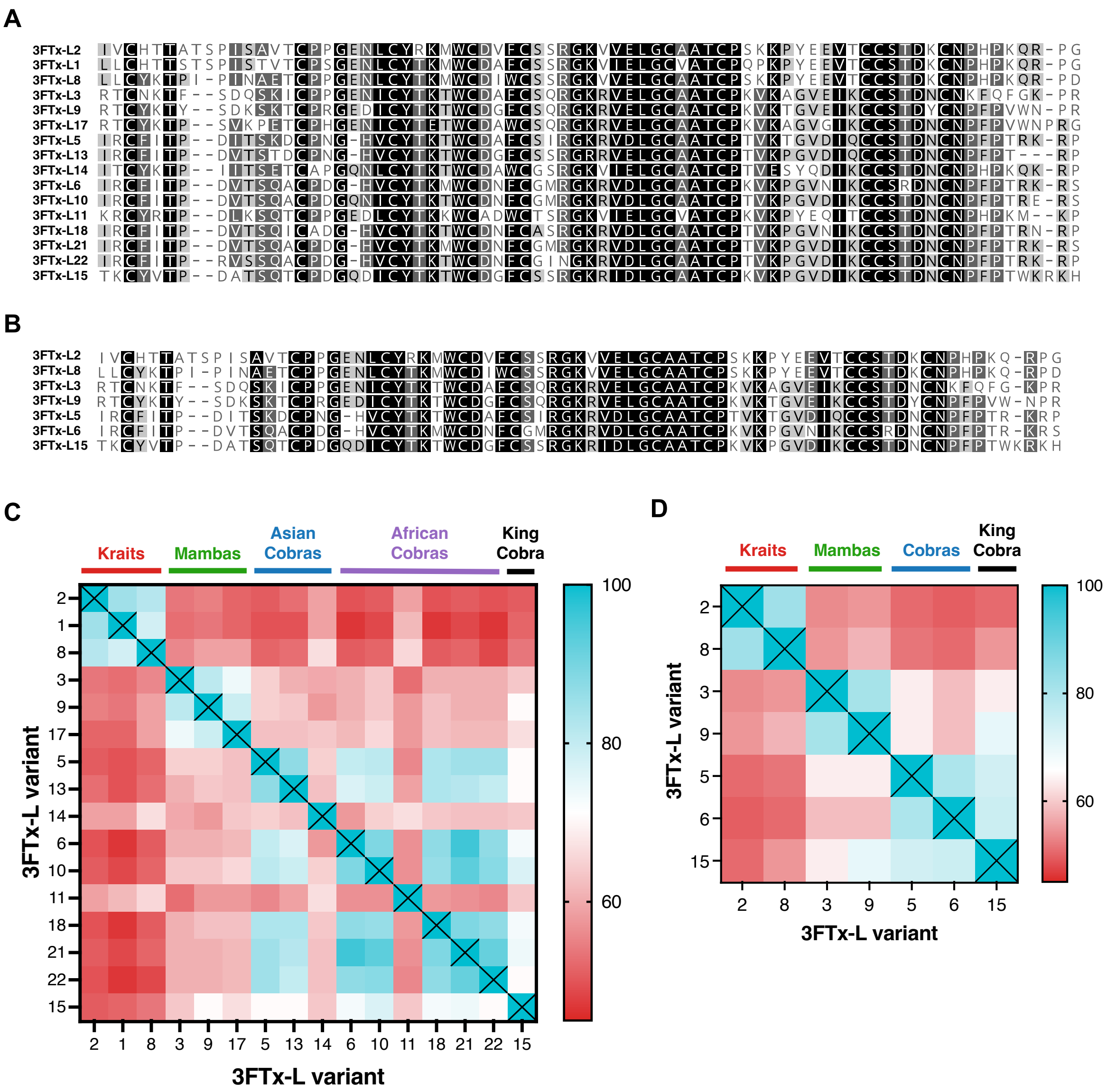

Fig. S1. Sequences of 3FTx-L variants selected for recombinant expression, functional validation, and characterization. (A and B) Sequence alignments of long-chain three-finger toxin (3FTx-L) variants from the full collection (**A**) and those used in the characterization panel (**B**). **(C and D)** The alignment distances (% sequence similarity between each variant) are shown plotted in heat map form for both the full collection (**C**) and the characterization panel (**D**).

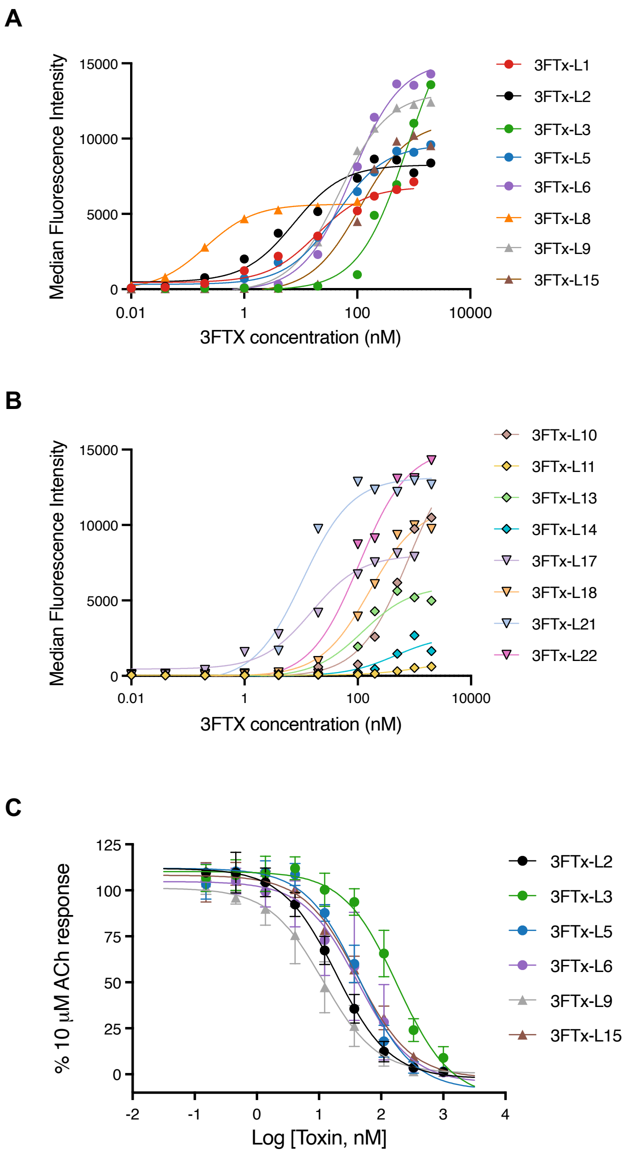

Fig. S2. Functional validation of recombinant 3FTx-L variants. (A and B) Binding curves for main (**A**) and supplemental (**B**) recombinant 3FTx-L variants on yeast-displayed human nicotinic acetylcholine receptor (nAChR) α1 subunit measured by flow cytometry. The median fluorescence intensity was plotted for each population of streptavidin-allophycocyanin (APC)-stained yeast cells displaying nAChRα1 and incubated with various concentrations of biotinylated 3FTx-L variants. Non-linear curve fitting was performed to calculate the half-maximal effective concentration (EC_{50}) for each variant using a one-site competitive binding model in Prism. (**C**) Concentration-inhibition plots demonstrating the antagonism of 3FTx-L variants from the characterization panel on the acetylcholine-induced activation of nAChRs expressed in TE671 cells. Each response was normalized to the control response (10 μM acetylcholine), and error bars represent the standard deviation of 3 replicate experiments.

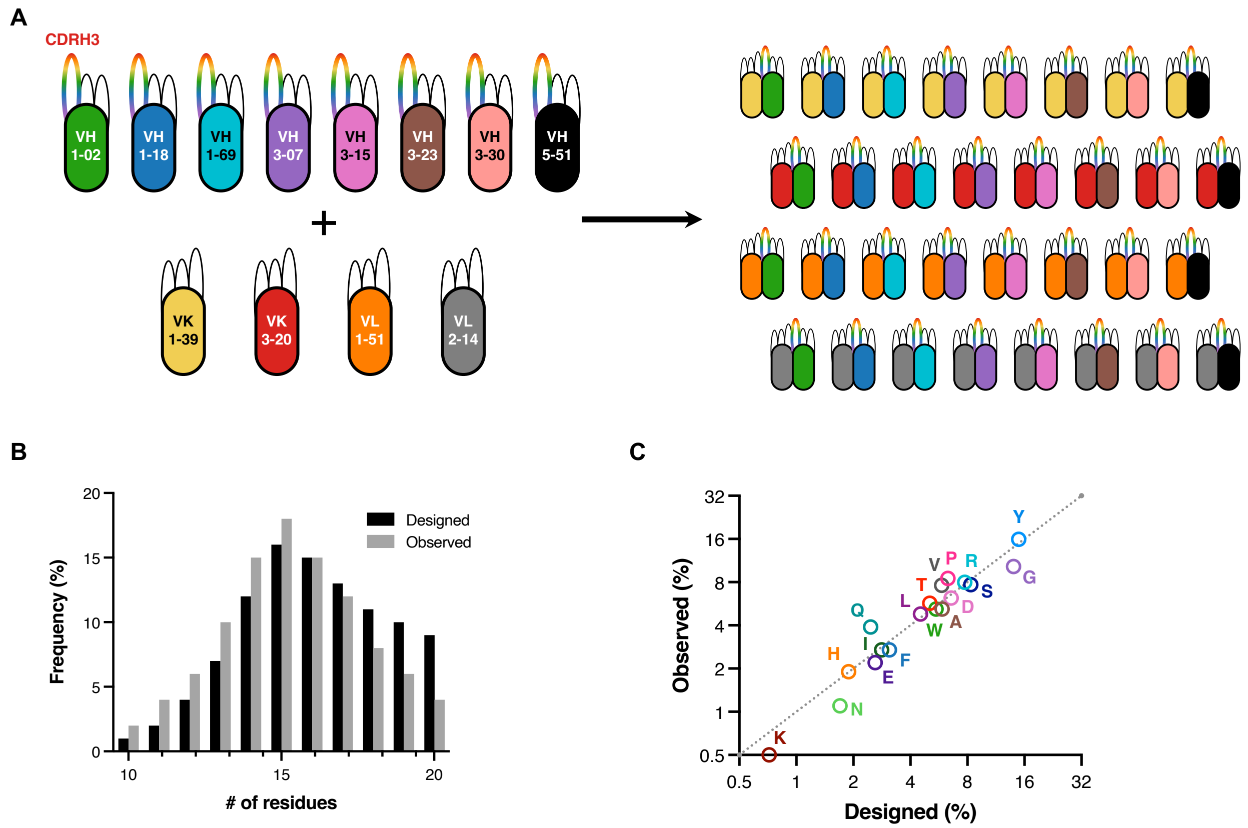

Fig. S3. Construction and validation of antibody library. (**A**) Fab library constructed using 8 different variable heavy chain (VH) domains containing synthetic diversity in the complementarity determining region 3 of the HC (CDRH3) paired with 4 universal variable light chain (VL) domains for a total of 32 different Fab combinations containing $6x10^{10}$ unique antibodies. **(B and C)** Designed versus observed frequencies of the CDRH3 lengths (**B**) and the amino acid residues (**C**) in the naive Fab library.

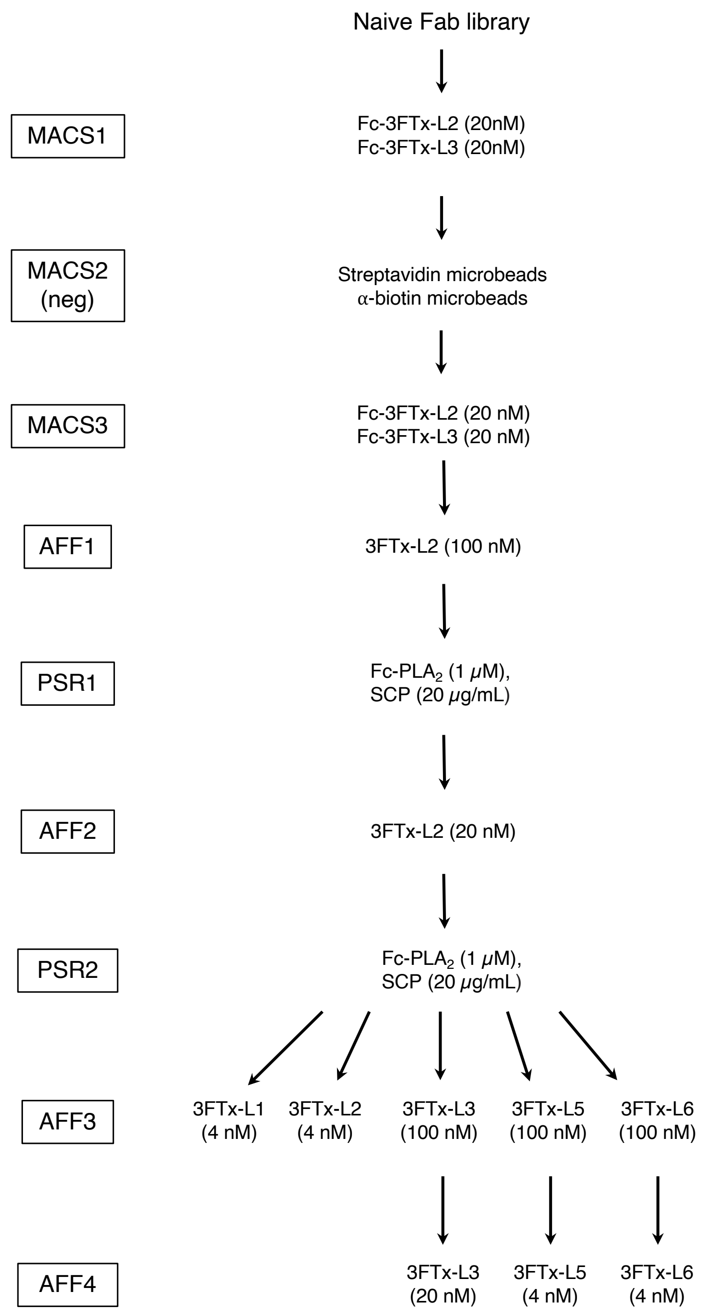

Fig. S4. Flow chart of sorts from the naive Fab library for isolation of cross-reactive anti-3FTx-L antibodies. Three rounds of MACS were performed initially with Fc-conjugated 3FTx (Fc-3FTx-L2 and Fc-3FTx-L3), followed by 5 rounds of FACS that were either affinity (AFF) sorts or negative (PSR) sorts. Negative sorting antigens included recombinant rabbit Fc-tagged phospholipase A_2 (Fc-PLA_2) and soluble cytosolic protein extract (SCP). The concentration of antigen used is indicated in parentheses. See the Methods section for further details.

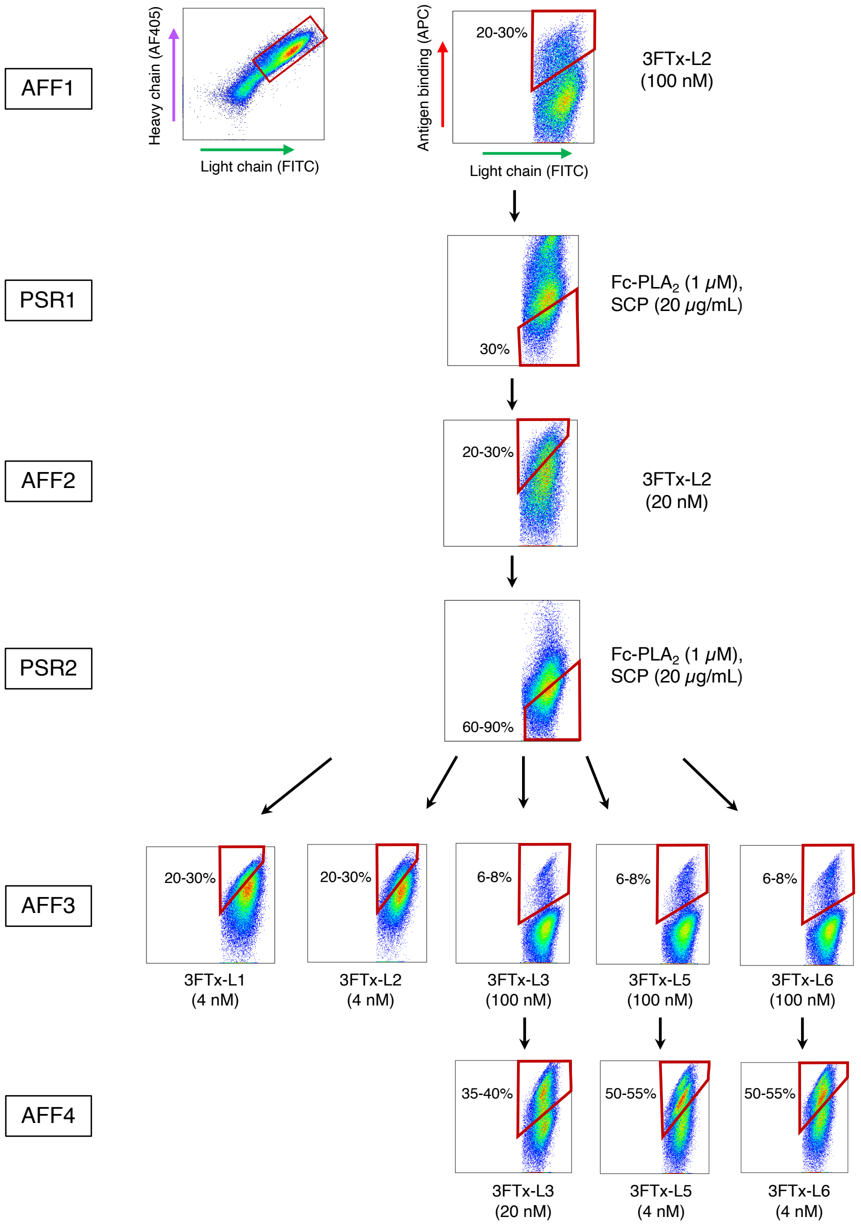

Fig. S5. Sorts from the naïve Fab library for isolation of cross-reactive anti-3FTx-L antibodies. Flow cytometry plots for each anti-3FTx sorting step showing the population of cells sorted for each round of FACS described in **fig. S4**. An example of paired chain selection is shown for AFF1, gating the population of Fabs with coordinated HC versus LC display signals. Antigen-binding or non-binding cells were then sorted in the AFF or PSR sorts, respectively, as shown by the gates drawn with red outlines. The percentage of the paired chain population that was sorted is indicated next to the sorted population, and the concentration of antigen used is indicated in parentheses. AF, Alexa Fluor; FITC, fluorescein isothiocyanate.

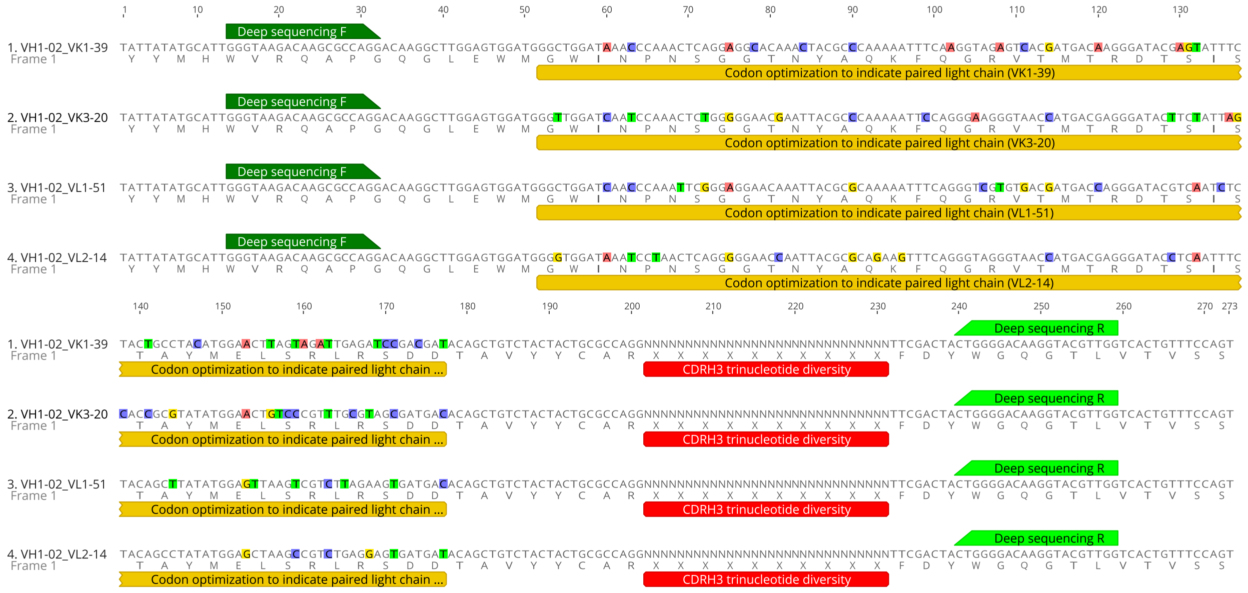

Fig. S6. Example of amplicon used for deep sequencing of Fab library. 4 different codon-optimized versions of VH1-02 containing nucleotide barcodes were used for identification of the paired LC. The barcodes were present across a 124 bp region upstream of the CDRH3, and the deep sequencing primers flanked both regions. Similar LC barcodes were applied to the other seven VH in the Fab library for a total of 32 HC/LC pairings.

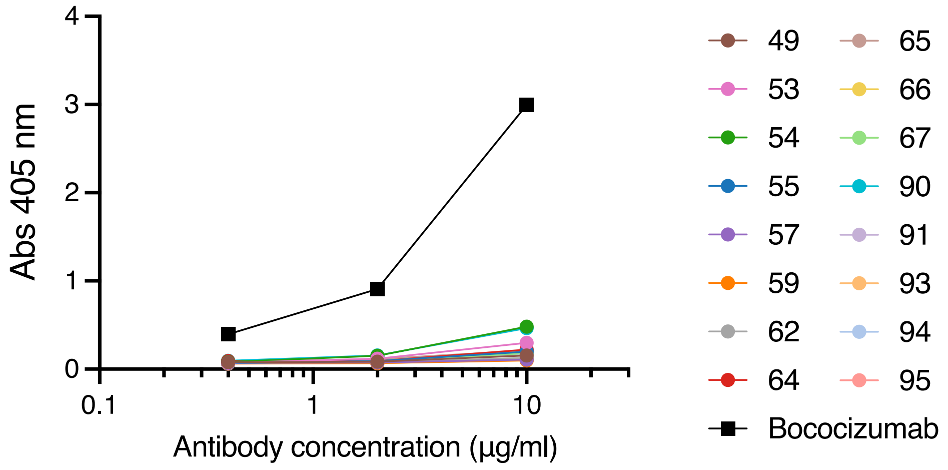

Fig. S7. Enzyme-linked immunosorbent assay (ELISA) screen for non-specific binding of cross-reactive 3FTx-L antibodies using cell soluble membrane protein extract (CHO-SMP). The sixteen 3FTx-L antibodies are compared against a polyreactive antibody (bococizumab) as a control. Abs, absorbance.

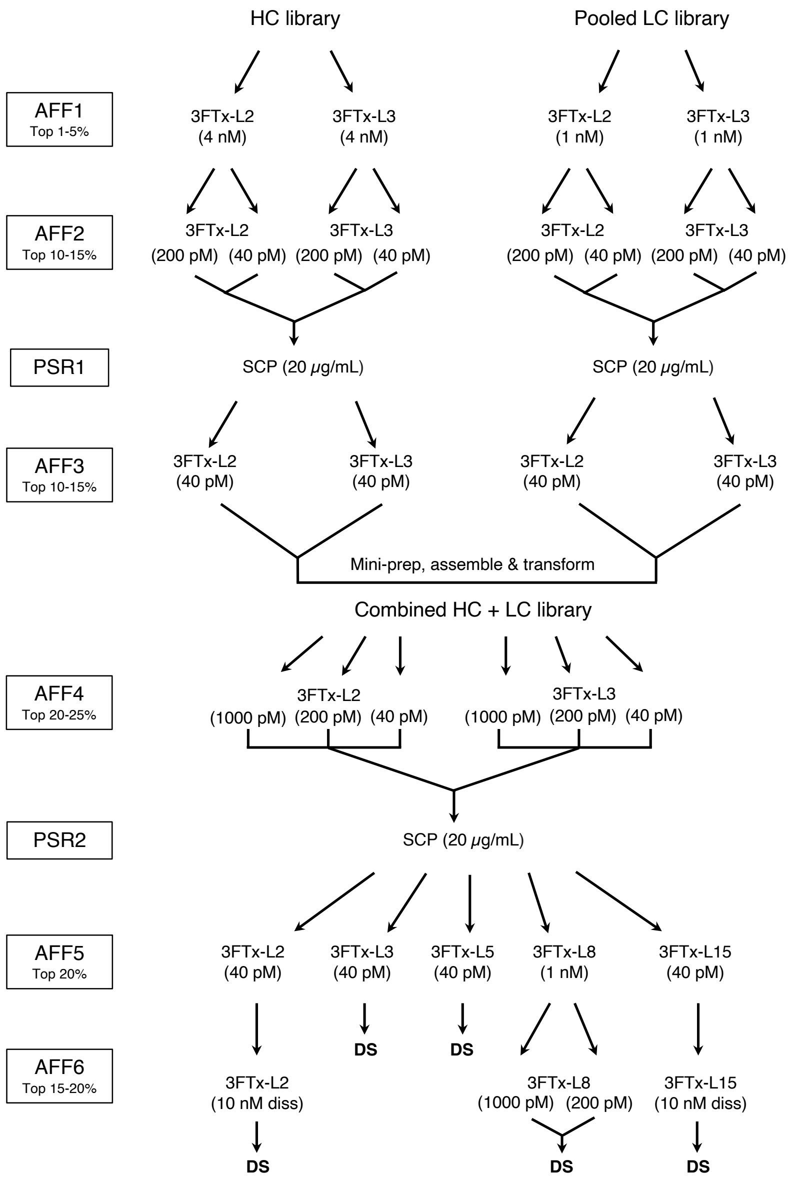

Fig. S8. Flow chart of sorts used in sorting the LB5_95 affinity maturation library. Four rounds of FACS were performed with the separate HC and LC libraries prior to combining them, followed by an additional 4 rounds of FACS with the combined library. The percentage of the paired chain population that was sorted is indicated under the name of each sort, and the concentration of antigen used is indicated in parentheses. Prior to each PSR sort, the cell populations from the previous affinity sort were combined before sorting with the SCP. Sorts with dissociation conditions applied are indicated with "diss" and sorts that were sent for deep sequencing are noted with "DS". See the Methods section for further details.

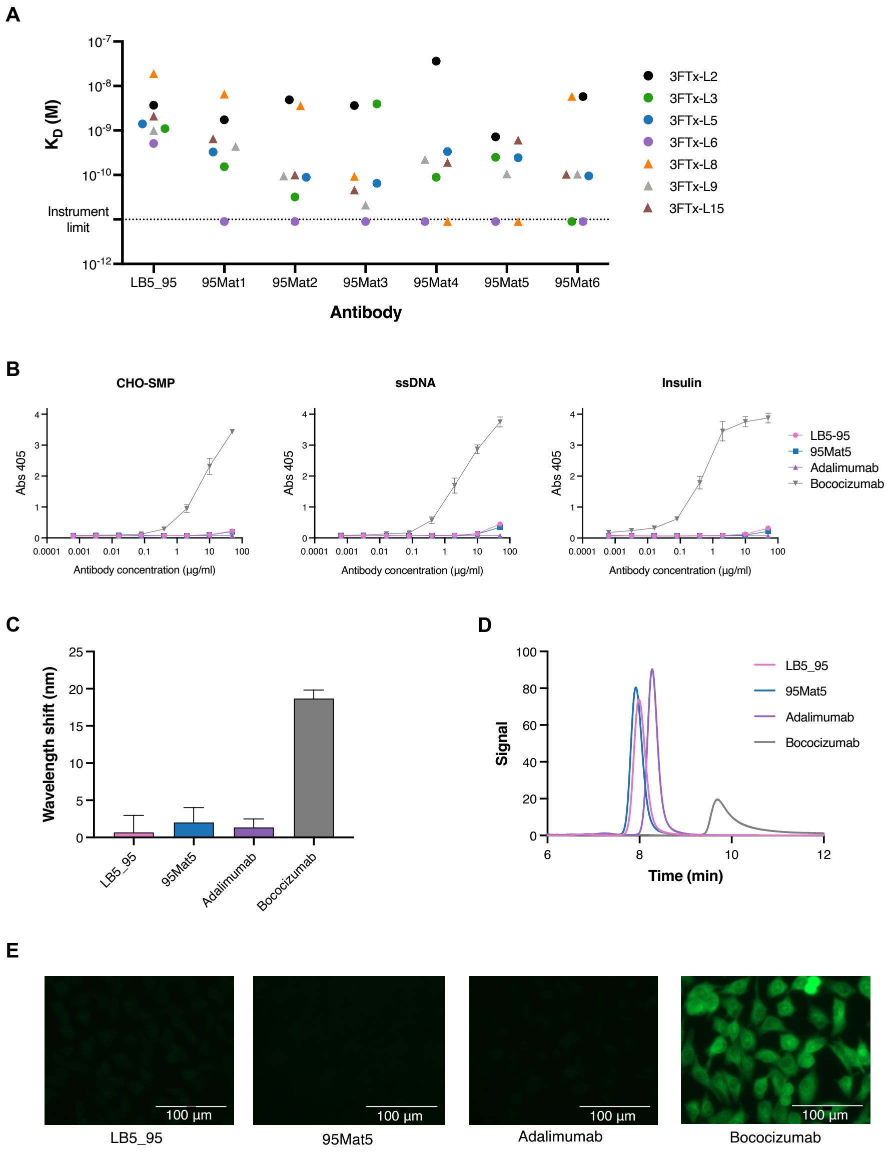

Fig. S9. Characterization of affinity matured antibodies. (**A**) Binding affinities for the 6 matured antibodies with the toxin panel variants in comparison to the parent antibody (LB5_95) as measured using surface plasmon resonance (SPR). K_D, dissociation constant. (**B**) ELISA to assess polyreactivity of antibodies on PSR reagents: CHO-SMP, single-stranded DNA (ssDNA), and insulin. LB5_95 and the lead matured antibody (95Mat5) are compared to adalimumab (good developability) and bococizumab (poor developability) controls. Error bars represent the standard deviation from 3 replicate experiments. (**C**) Affinity-capture self-interaction nanoparticle spectroscopy (AC-SINS) characterization of LB5_95 and 95Mat5 comparing wavelength shifts (nm) to control antibodies. (**D**) Analytical size exclusion chromatography performed for LB5_95 and 95Mat5 in comparison to controls. (**E**) Images of HEp-2 cells stained with antibodies in comparison to controls.

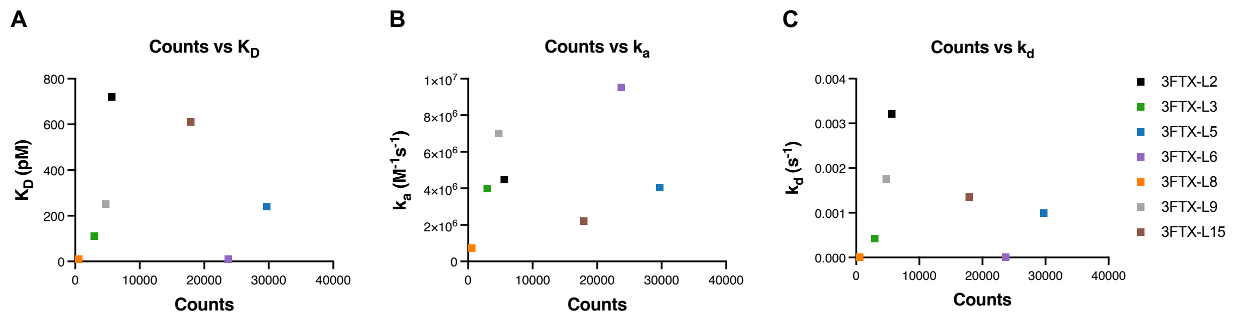

Fig. S10. Deep sequencing counts for 3FTx-L variants from yeast-displayed library sorted with 95Mat5 and plotted as a function of SPR affinity measurements. (A to C) Dissociation constants (K_D, **A**), association rates (k_a, **B**), and dissociation rates (k_d, **C**) are shown.

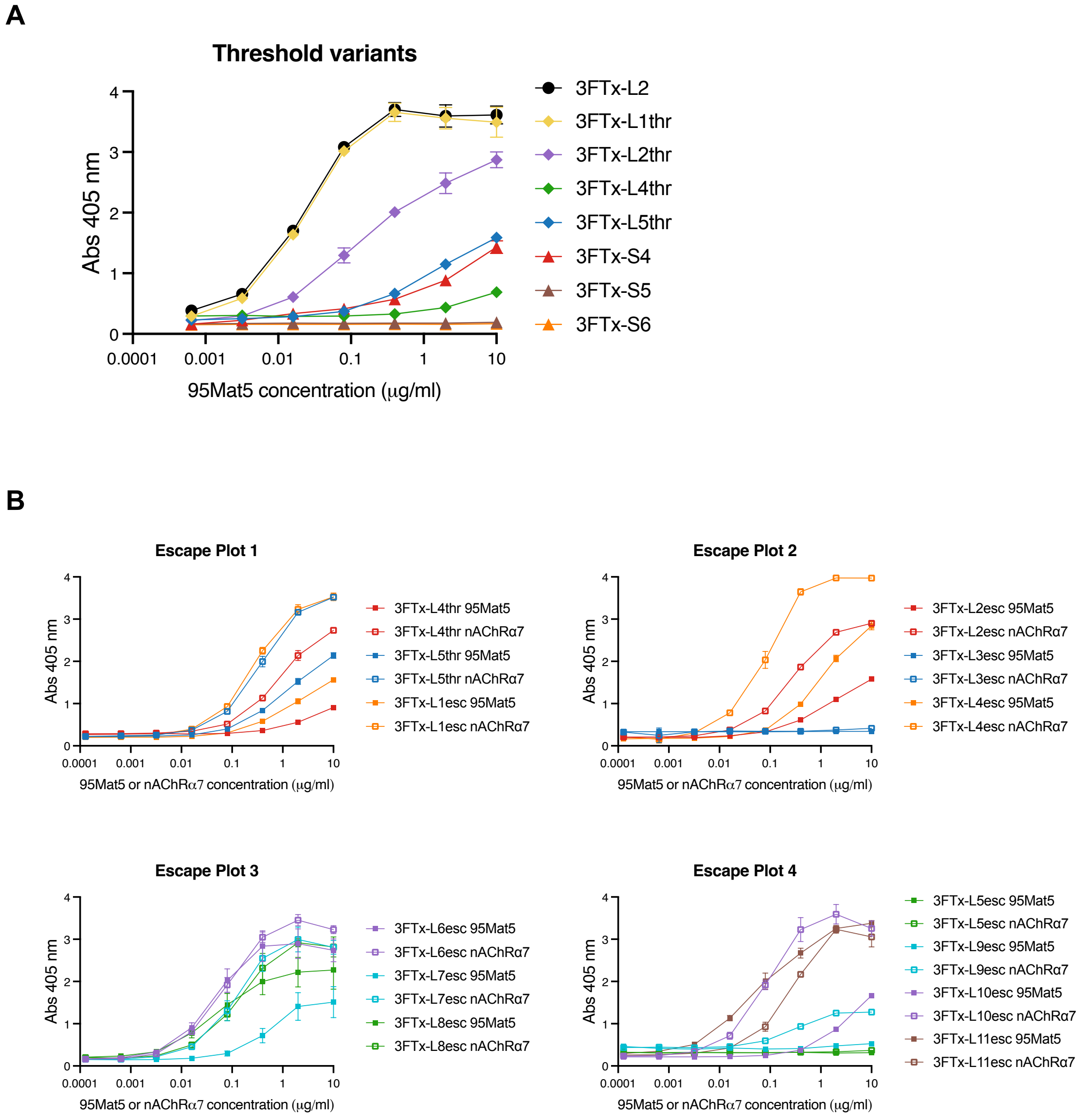

Fig. S11. Validation of 95Mat5-binding 3FTx variants at the deep sequencing threshold and potential escape variants. (**A**) ELISA results for 95Mat5 binding to expressed threshold 3FTx-L and 3FTx-S variants listed in **table S3** as compared with 3FTx-L2. (**B**) ELISA results for 95Mat5 binding versus nAChRα7 binding to potential 3FTx-L escape variants identified by deep sequencing. Error bars represent the standard deviation from 3 replicate experiments.

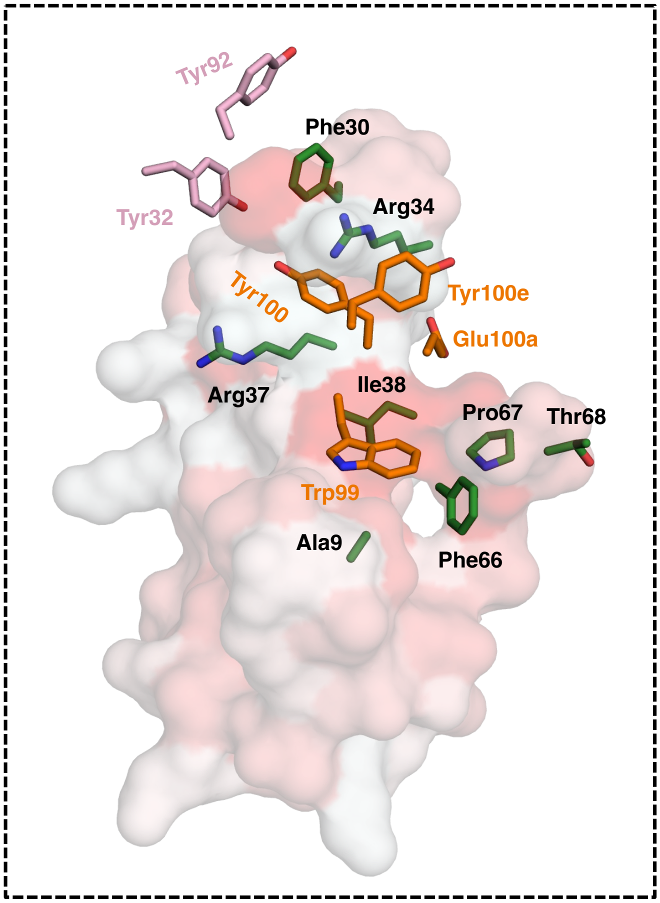

Fig. S12. 95Mat5 Fab recognizes hydrophobic pockets of 3FTx-L15. 3FTx-L15 is presented by a hydrophobic surface with red for high degrees of hydrophobicity and whitish gray for high degrees of hydrophilicity.

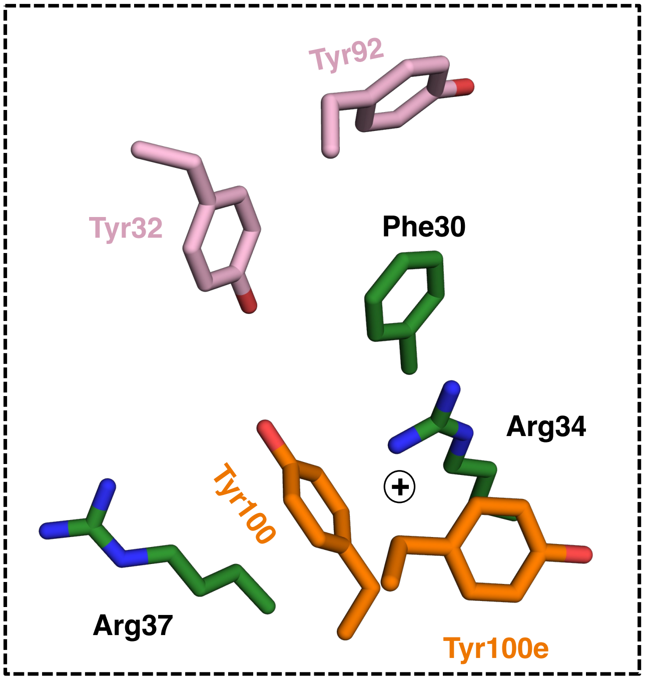

Fig. S13. The 95Mat5:3FTx-L15 interface involves a cation/amino-π interaction. 3FTx-L15, 95Mat5 HC, and 95Mat5 LC residues are shown in green, orange, and pink sticks. Two cation/amino–π interactions involve Tyr100 with Arg34 and Arg34 with Phe30.

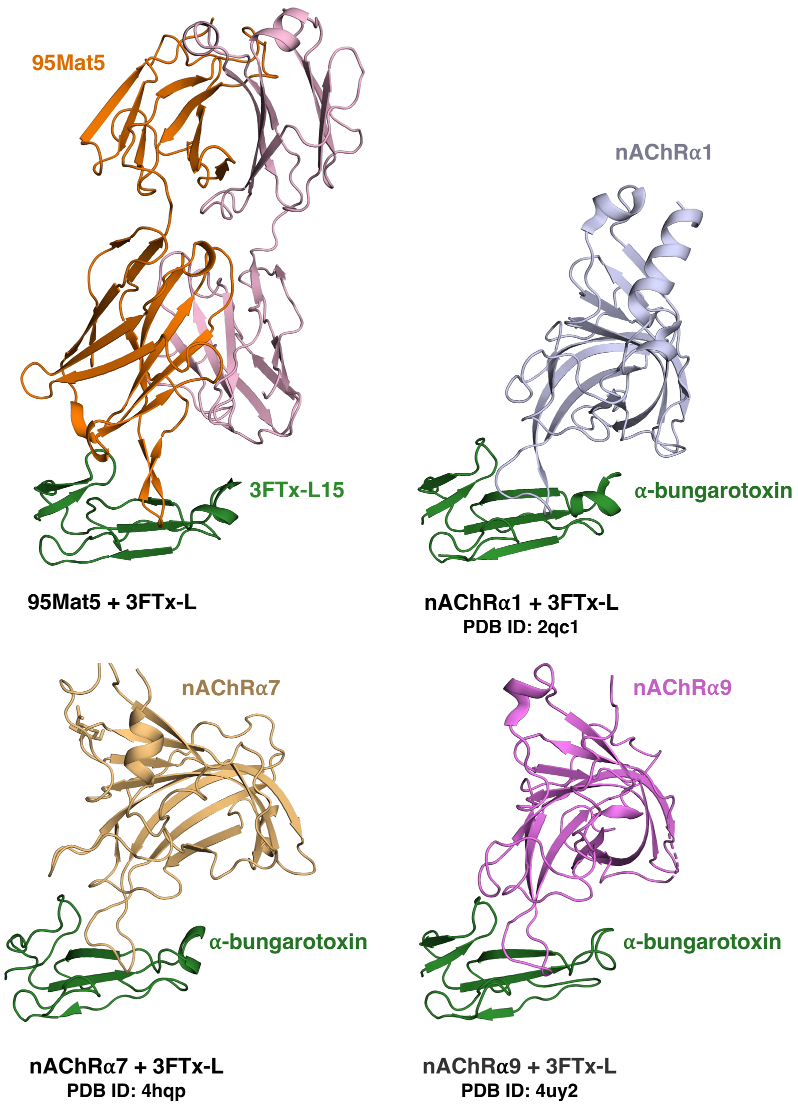

Fig. S14. Comparison of the binding mode for 95Mat5:3FTx-L15 with multiple nAChRα subunits in complex with 3FTx-L. 3FTx-L is in dark green cartoon representation. Human nAChRα1, α7, and α9 are shown in lavender, gold, and magenta cartoons, respectively.

Table S1. List of recombinant 3FTx-L variants synthesized and tested for nAChRα1 binding. UniProt and PDB accession numbers are provided along with any names associated with the toxin variants, snake species, and geographic regions. The half-maximal effective concentration (EC_{50}) measured from the curves in **fig. S2** for binding to yeast-displayed human nAChRα1 subunit is indicated for each variant. DNB, did not bind; UND, undetermined.

Variant name	Accession	Other name	Species	Common name	Region	nACRα1 EC_{50}
3FTx-L1	D2N116	Alpha-delta bungarotoxin-4	*Bungarus caeruleus*	Common krait	South central Asia	19 nM
3FTx-L2	P60616, 1HC9	Alpha-bungarotoxin V31	*Bungarus multicinctus*	Many-banded krait	Southeastern Asia	7.5 nM
3FTx-L3	C0HJD7, 4LFT	Alpha-elapitoxin-Dpp2d	*Dendroaspis polylepis*	Black mamba	East central & southern Africa	UND
3FTx-L5	P01391, 2CTX, 4AEA	Alpha-cobratoxin	*Naja kaouthia, Naja siamensis*	Monacled cobra, Indochinese spitting cobra	Southeastern Asia	42 nM
3FTx-L6	P01390	Neurotoxin alpha	*Naja nivea*	Cape cobra	Southern Africa	81 nM
3FTx-L8	A1IVR9	Alpha-elapitoxin-Bc2b	*Bungarus candidus*	Malayan krait	Southern Asia	0.21 nM
3FTx-L9	P01393	Alpha-elapitoxin-Djk2a	*Dendroaspis jamesoni*	Jameson's mamba	West central Africa	49 nM
3FTx-L10	P01389	Toxin III	*Naja anchietae*	Anchieta's cobra	South central Africa	UND
3FTx-L11	P01383	Neurotoxin 3.9.4	*Naja melanoleuca*	Forest cobra	West central Africa	DNB
3FTx-L13	O42257	Long neurotoxin 7	*Naja sputatrix*	Javan spitting cobra	Indonesia	122 nM
3FTx-L14	1NTN	Neurotoxin 1	*Naja oxiana*	Caspian cobra	Central Asia	DNB
3FTx-L15	P01386, 1TXA	Toxin B	*Ophiophagus hannah*	King cobra	Southeastern Asia	113 nM
3FTx-L17	P01395	Alpha-elapitoxin-Dv2a	*Dendroaspis viridis*	Western green mamba	Western Africa	15 nM
3FTx-L18	P01388	Neurotoxin B	*Naja melanoleuca*	Forest cobra	West central Africa	162 nM
3FTx-L21	P25674	Toxin CM-5	*Naja haje*	Egyptian cobra	Northern Africa	11 nM
3FTx-L22	P34074	Long neurotoxin 1	*Naja annulata*	Banded water cobra	West central Africa	106 nM

Table S2. SPR measurements for LB5-95 binding with 3FTx-L variants. Dissociation constants (K_D), association rates (k_a), and dissociation rates (k_d) are shown.

Variant name	k_a ($M^{-1}s^{-1}$)	k_d (s^{-1})	K_D (M)
3FTx-L2	$2.3E^{6}$	$8.7E^{-3}$	$3.7E^{-9}$
3FTx-L3	$1.5E^{6}$	$1.7E^{-3}$	$1.1E^{-9}$
3FTx-L5	$4.5E^{6}$	$6.5E^{-3}$	$1.4E^{-9}$
3FTx-L6	$4.1E^{6}$	$2.1E^{-3}$	$5.1E^{-10}$
3FTx-L8	$5.6E^{5}$	$1.0E^{-2}$	$1.9E^{-8}$
3FTx-L9	$3.0E^{6}$	$3.1E^{-3}$	$1.0E^{-9}$
3FTx-L15	$4.9E^{6}$	$1.0E^{-2}$	$2.1E^{-9}$

Table S3. List of recombinant 3FTx-L and 3FTx-S library variants selected from deep sequencing data that were synthesized and tested for binding to 95Mat5 or nAChRα7 in fig S11. Variant names are listed as they appear in **data file S3** along with species, corresponding UniProt accession numbers, and number of sequencing counts in the sum of the final sorts with 95Mat5. Threshold variants had total counts near or slightly below 100 in the final sort with 95Mat5 whereas potential escape variants were enriched in the final nAChRα sorts but not in the final antibody sort. The outcome of the ELISA results or the inability of the variant to express from mammalian cells is listed in the final column. Dysfunctional variants were non-binding to both 95Mat5 and nAChRα7 whereas escape variants exhibited higher binding to nAChRα7 versus 95Mat5 (**fig S11B**).

Class	Designation	Variant name	Species	Accession	Counts	Outcome
Long alpha threshold	3FTx-L1thr	Alpha-bungarotoxin (T7/A8)	*Bungarus candidus*	CAM11306	472	mAb-binding
	3FTx-L2thr	AUSLA putative long neurotoxin 5	*Austrelaps labialis*	ABX58157	116	mAb-binding
	3FTx-L3thr	OPHHA 256	*Ophiophagus hannah*	T.D. Kazandijian et al., *Science* (2021)	98	Did not express
	3FTx-L4thr	AUSLA putative long neurotoxin 11	*Austrelaps labialis*	ABX58163	76	Escape
	3FTx-L5thr	AUSLA putative long neurotoxin 12	*Austrelaps labialis*	ABX58164	72	Escape
Long alpha escape	3FTx-L1esc	Long neurotoxin LlLong	*Laticauda laticaudata*	Q7T2I3	53	Escape
	3FTx-L2esc	NAJME Neurotoxin 3.9.4	*Naja melanoleuca*	P01383	48	Escape
	3FTx-L3esc	Putative long chain neurotoxin 472	*Drysdalia coronoides*	ACR78488	39	Dysfunctional
	3FTx-L4esc	Alpha-bungarotoxin isoform R13	*Bungarus multicinctus*	AAC83995	13	Escape
	3FTx-L5esc	Putative long chain neurotoxin 291	*Drysdalia coronoides*	ACR78483	11	Dysfunctional
	3FTx-L6esc	Alpha-bungarotoxin K36	*Bungarus multicinctus*	AAC83982	Not present	mAb-binding
	3FTx-L7esc	Alpha-bungarotoxin isoform R5	*Bungarus multicinctus*	AAC83986	Not present	Escape
	3FTx-L8esc	Long neurotoxin 43	*Drysdalia coronoides*	F8J2D7	Not present	mAb-binding
	3FTx-L9esc	Long neurotoxin LNTX28	*Ophiophagus hannah*	Q2VBP4	Not present	Dysfunctional
	3FTx-L10esc	Long neurotoxin LNTX-1	*Demansia vestigiata*	A6MFK4	Not present	Escape
	3FTx-L11esc	Putative long chain neurotoxin 346	*Drysdalia coronoides*	ACR78479	Not present	mAb-binding
Short alpha	3FTx-S1	MICAL MALT0057C	*Micrurus altirostris*	F5CPD8	10681	Did not express
	3FTx-S2	MICDI three finger toxin precursor D.J.	*Micrurus diastema*	AKN63197	1401	Did not express
	3FTx-S3	MICCO 3FTx precursor	*Micrurus corallinus*	ACS74994	542	Did not express
	3FTx-S4	NAJNA cobratoxin	*Naja naja*	Q9PTT0	214	Weak binding
	3FTx-S5	DENJA Toxin S5C10	*Dendroaspis jamesoni*	P01419	92	Non-binding
	3FTx-S6	DENJA T0913	*Dendroaspis jamesoni*	S. Ainsworth et al., *J. Proteomics* (2018)	53	Non-binding

Table S4. **Intravenous and subcutaneous median lethal dose (LD_{50}) measured for α-bungarotoxin and crude venoms.**

Toxin/venom	Intravenous LD_{50}	Subcutaneous LD_{50}
α-bungarotoxin	10.8 µg/mouse (0.541 mg/kg)	Not tested
Naja kaouthia	10.7 µg/mouse (0.536 mg/kg)	13.8 µg/mouse (0.688 mg/kg)
Ophiophagus hannah	17.9 µg/mouse (0.890 mg/kg)	21.5 µg/mouse (1.08 mg/kg)
Dendroaspis polylepis	6.99 µg/mouse (0.349 mg/kg)	7.17 µg/mouse (0.358 mg/kg)

Table S5. Neutralization potencies and dosages of monovalent antivenoms marketed by Queen Saovabha Memorial Institute (QSMI) for treating *N. kaouthia* and *O. hannah* envenoming, and African polyvalent antivenom marketed by Premium Serums and Vaccine Pvt. Ltd. (PSVPL) for treating *D. polylepis* bites. The marketed neutralization potency for each commercial antivenom is listed in units of mg venom that could be neutralized by 1 ml of undiluted antivenom, whereas the antivenom concentration is listed in units of mg serum protein (based on the dry weight of antivenom product contained in a vial) per ml of reconstituted antivenom solution. The antivenom dosage is then converted to mg of serum protein per kg of animal weight.

Manufacturer	Batch number	Species	Type	Venom dosage	Antivenom potency	Antivenom concentration	Antivenom dosage
QSMI	NK00112	*Naja kaouthia*	Monovalent	2x LD_{50}: 22 µg/mouse	0.6 mg/ml	61.9 mg/ml	113 mg/kg
PSVPL	PANAF-016	*Dendroaspis polylepis*	Polyvalent	2x LD_{50}: 14 µg/mouse	0.175 mg/ml	95.3 mg/ml	381 mg/kg
QSMI	LH00112	*Ophiophagus hannah*	Monovalent	2x LD_{50}: 36 µg/mouse	0.8 mg/ml	42.2 mg/ml	95.0 mg/kg

Table S6. Data collection and refinement statistics of 3FTx-L15 and 95Mat5 Fab complex structure.

**Data Collection**	
X-ray source:	Rigaku MicroMax-007
Wavelength (Å)	1.5418
Space group	C121
Cell dimensions	
a, b, c (Å)	135.86, 42.44, 103.36
α, ß, γ (°)	90, 103.7, 90
Resolution D_{min} (Å)[a]	60.00-2.90 (2.95-2.90)
Effective Resolution D_{eff}[b] (Å)[a]	60.00-3.09
R_{sym}[a,c]	0.08 (0.42)
R_{pim}[a,d]	0.06 (0.35)
Unique reflections	10,753
Average I/σ(I)[a]	17.8 (0.9)
$CC_{1/2}$[e]	0.99 (0.84)
Completeness (%)[a]	82.1 (14.9)
Redundancy[a]	2.7 (1.5)
**Refinement Statistics**	
Resolution range (Å)	45.53-2.90
Reflections in refinement	10,741
R_{cryst}[f]	0.26
R_{free}[g]	0.32
No. atoms	
3FTx-L15	503
95Mat5 Fab	3,332
Water	3
Wilson B-value (Å^2)	83
Average B-values (Å^2)	
3FTx-L15	118
95Mat5 Fab	87
Water	29
R.M.S. Deviations	
Bond length (Å)	0.013
Bond angles (°)	2.04
Ramachandran statistics (%)[h]	
Favored/outlier	92.23 (0.00)
Number of TLS groups	1

[a] Value s in parentheses denote outer-shell statistics.

[b] $D_{eff} = D_{min} * C\text{^} (-1/3)$ where C is Completeness (%)

[c] $R_{sym} = \sum_{hkl}\sum_i |I_{hkl,i}$ - | $/\sum_{hkl}\sum_i I_{hkl,i}$ and $R_{pim} = \sum_{hkl}[1/(N-1)]1/2\sum_i$ $|I_{hkl,i}$ - | $/\sum_{hkl}\sum_i I_{hkl,i,}$ where $I_{hkl,i}$ is the scaled intensity of the ith measurement of reflection h, k, l, < I_{hkl}> is the average intensity for that reflection, and N is the redundancy.

[d] $R_{pim} = \Sigma_{hkl}$ (1/(n-1))1/2 Σ_i | $I_{hkl,i}$ - | / Σ_{hkl} Σ_i $I_{hkl,i}$, where n is the redundancy.

[e] $CC_{1/2}$ = Pearson correlation coefficient between two random half datasets.

[f] $R_{cryst} = \sum_{hkl}$ |Fo - Fc| / $\sum_{hkl}$ |Fo|, where Fo and Fc are the observed and calculated structure factors.
[g] R_{free} was calculated as for R_{cryst}, but on 5% of data excluded before refinement.

[h] The values are percentage of residues in the favored and outlier regions analyzed by MolProbity (*71*).

Table S7. Interactions between 95Mat5 and 3FTx-L15 by PISA web server.

	Hydrogen Bonds			Ionic Interactions		
	95Mat5	Distance (Å)	3FTx-L15	95Mat5	Distance (Å)	3FTx-L15
CDRH1	Thr28 [OG1]	3.2	Asp8 [OD1]			
	Ser31 [OG]	4.4	Pro7 [O]			
	Ser31 [OG]	3.3	Asp8 [OD1]			
CDRH3				Arg98[NH1]	5.3	Asp28[OD2]
	Trp99 [NE1]	3.6	Pro7 [O]			
	Trp99 [O]	2.8	Ile38 [N]			
	Glu100a [N]	3.7	Arg34 [O]			
	Ser100b [N]	3.3	Arg34 [O]			
	Glu100a [N]	2.9	Lys36 [O]	Glu100a OE2]	3.2	Lys36 [NZ]
	Tyr100 [OH]	3.1	Arg34 [NH1]			
	Tyr100 [OH]	2.8	Asp28 [OD2]			
	Glu100a [OE1]	2.8	Lys36 [N]			
	Glu100f [O]	4.2	Arg34 [NH1]			
CDRL1	Tyr32 [OH]	3.6	Phe30 [N]			
	Tyr32 [OH]	2.9	Asp28 [OD2]			
	Tyr32 [OH]	3.6	Gly29 [N]			
CDRL2				Asp50 [OD1]	4.9	Arg37 [NH1]

Due to the relatively low resolution of the structure, generous distances for potential hydrogen bonds and ionic interactions are used.

Table S8. Contacts between nAChRα1:α-bungarotoxin (PDB: 2QC1) and 95Mat5:3FTx-L15.

	nAChRα1	α-bungarotoxin
**Loop C**	Trp187	Ser9
	Val188	Val39
	Phe189	Thr6, Thr8, Ser9, Ile11, Val39, **Val40**
	Tyr190	**Asp30**, Phe32, **Arg36**, Gly37, Lys38, Val39, Val40, His68
	Ser191	**Arg36, Lys38**, Val40, **His68**, Pro69, Lys70
	Cys192	**Arg36**, **Lys70**
	Cys193	His68
	Pro194	His68, Lys70, Gln71
	Pro197	Ser9
	Tyr198	Arg36
**Loop B**	Thr148	**Arg36**
	Arg149	Phe32, **Arg36**
**Loop A**	Val91	Phe32
	Tyr93	Val31, Phe32
	Asp99	Val31
	Phe100	Val31, Phe32
	**95Mat5**	**3FTx-L15**
**95Mat5 heavy chain**	Thr28	**Asp8**
	Ser31	**Pro7, Asp8**
	Tyr32	Pro7
	Arg98	**Asp28**, Arg37
	Trp99	Thr6, **Pro7**, Ala9, Arg37, **Ile38**, Phe66, Thr68
	Tyr100	**Asp28**, Phe30, **Arg34**, Gly35, Lys36, Arg37, Ile38
	Glu100a	**Arg34, Lys36**, Ile38, Pro67
	Ser100b	**Arg34**, Thr68
	Gly100c	Thr68
	Tyr100e	Arg34
	Glu100f	**Arg34**
**95Mat5 light chain**	Tyr32	**Asp28, Gly29**, **Phe30**
	Asp50	**Arg37**
	Ser91	Phe30
	Tyr92	Phe30

3FTx-L15 and α-bungarotoxin residues involved in H-bonds and ionic interactions are shown in bold; van der Waals interactions are in the normal font; hydrophobic interactions are underlined.

Data file S1. Deep sequencing results for the anti-3FTx-L2 Fabs used in **Fig. 1** cross-reactive analysis.

Data file S2. Sequences for cross-reactive antibodies against 3FTx-L.

Data file S3. Deep sequencing results for the 3FTx library sorted with 95Mat5. The variant names are color-coded based on 3FTx family using the same convention as in **Fig. 3A**, and the nucleotide and protein sequences are provided for each codon version. The sum of counts from the top 2 codon versions was used to rank the enrichment for each variant in each sort. The sum of counts from the bottom 2 codon versions was used to filter out variants that were enriched with only one codon version in each sort. For the third sort with 95Mat5, additional sorts were performed for high affinity (PopA), moderate affinity (PopB), and low affinity (PopC) binders, and the sum of the counts from all four populations (all binders, PopA, PopB, and PopC) was used to rank enrichment.

Data file S4. Deep sequencing results for the 3FTx library sorted with nAChRα1. The sum of counts from the top 2 codon versions was used to rank the enrichment for each variant in each sort. The sum of counts from the bottom 2 codon versions was used to filter out variants that were enriched with only one codon version in each sort.

Data file S5. Deep sequencing results for the 3FTx library sorted with nAChRα7. The sum of counts from the top 2 codon versions was used to rank the enrichment for each variant in each sort. The sum of counts from the bottom 2 codon versions was used to filter out variants that were enriched with only one codon version in each sort.

Data file S6. List of the 149 3FTx-L variants present in the library and their binding status for 95Mat5, nAChRα1, and nAChRα7 as determined by deep sequencing screening and ELISA testing of the variants listed in **table S2**. The sum of the counts in the final sorts with 95Mat5 are provided for each variant along with UniProt accession numbers or references, and snake species. Synthesized variants are also identified with their study designations and notes on how their binding results were determined.

Data file S7. Raw, individual-level data for experiments where $n<20$.
